# Deep learning based SentiNet architecture with hyperparameter optimization for sentiment analysis of customer reviews

**DOI:** 10.1038/s41598-025-19532-3

**Published:** 2025-10-10

**Authors:** B. Madhurika, D. Naga Malleswari

**Affiliations:** Koneru Lakshmaiah Education Foundation, Vaddeswaram, Andhra Pradesh 522302 India

**Keywords:** Sentiment analysis, Opinion mining, Deep learning, Artificial intelligence, Online review analysis, Engineering, Mathematics and computing, Computer science, Information technology, Software

## Abstract

Due to the ever-increasing volume of opinionated content on social media, there is a pressing need for a highly effective process to perform sentiment analysis (NLP) successfully. But, methods nowadays are usually not good enough in context, long-term dependency, and domain-specific areas modelling, especially they lack in modelling of noisy, short texts. In this paper, we introduce SentiNet, a new hybrid deep learning architecture combining multi-layered BiLSTM encoders with convolutional feature extractors and an attention-based fusion mechanism, to achieve better performance in sentiment classification across various datasets. Our model incorporates embedding vectors, parallel convolutional layers, and channel-wise attention over embedded sequential data, as well as one or two bidirectional LSTM units to capture context over the sequence elements. We conduct extensive experiments on the IMDb, Twitter, and Yelp datasets, and show SentiNet yields the highest accuracy of 94.2%, best F1-score of 92.8%, and a balanced precision-recall curve, outperforming competitive baselines. To validate the contribution of each module, ablative experiments are performed, and cross-domain evaluations prove its robustness. The key contribution of this work is that it balances accuracy and interpretability within an efficient processing pipeline, making it applicable in real-world sentiment scenarios, such as e-commerce, customer experience monitoring, and social media analytics. This work extends scalable sentiment analysis by introducing a high-performing, interpretable, and adaptable framework, providing a strong foundation for future explainable AI advancements in text analytics. To enable reproducibility and follow-up work, the code and model will be released publicly.

## Introduction

Nowadays, individual decisions are increasingly influenced by social media and reviews from various online platforms regarding services and products. Businesses globally have recognized the importance of analyzing customer sentiments on these virtual platforms beyond traditional feedback systems. If companies fail to consider social feedback, particularly consumer sentiment expressed in reviews, they risk becoming irrelevant in today’s digital landscape. Thus, sentiment analysis of customer review data is essential for understanding customers’ thought processes. This understanding can help businesses adapt their products and services to meet customer expectations. Many researchers have contributed to applying both DL and ML techniques to sentiment analysis. For instance, Wang et al.^1^ introduced a Convolutional Recurrent Neural Network that combines LSTM networks with CNN to enhance text categorization accuracy. Said et al.^2^ employed Bi-LSTM-CRF and AB-LSTM-PC models to analyze Arabic hotel reviews, aiming to strengthen sentiment detection by incorporating external lexicons and Gated Recurrent Units (GRU). Sulaiman et al.^3^ emphasized the need for specialized techniques to analyze sentiment on Twitter, proposing a CNN model that integrates user behavior to improve classification on SemEval-2016 datasets. Jelodar et al.^4^ achieved an accuracy of 81.15% in analyzing public sentiment related to COVID-19 using LSTM and Natural Language Processing (NLP). Basiri et al.^5^ developed the ABCDM model, incorporating CNN layers and attention mechanisms, demonstrating solid results on tweets and reviews with potential for broader applications. It is clear from the literature research that artificial intelligence-enabled methods can significantly improve the identification of sentiments. However, there is a need to develop a hybrid methodology that combines the strengths of word embeddings, effectively processes time series data, and achieves accurate sentiment analysis within the given text corpus.

Despite the success of various state-of-the-art deep learning models for sentiment analysis, the majority of current methods fail to accurately capture the subtle sentiments in informal, short texts, especially under domain shift, context confusion, and multi-aspect sentiment expressions. Most models concentrate on either visual characteristics using CNNs or temporal information using RNNs, without an efficient fusion between the two. Furthermore, their interpretability is very poor, making them unsuitable for real-world, real-time applications, where both strings must be processed and analyzed simultaneously. To address the above limitations, this study proposes SentiNet. This unified deep learning architecture synergistically combines CNN-based local pattern extraction, BiLSTM-based temporal modeling, and an attention-mediated fusion process, designed to bridge the gaps in contextual sentiment comprehension, explainability, and cross-domain transfer. To meet the above challenges, the objectives of this research are as follows:

1. To develop an end-to-end sentiment classifier system which can successfully integrate local n-grams with global interactions in local multiple contexts using a convolutional, as well as a bi-directional recurrent structure;
2. To add a semantic attention layer to weight contextual and sentiment-bearing tokens for better interpretability dynamically, and to use a related non-linear function for prediction, thereby improving overall performance;
3. To test the model on multiple benchmark datasets and show its generalization and robustness against existing approaches; and.
4. To perform ablation and qualitative analyses to verify the contribution of each module and transparently generate sentiment prediction.

Here are the things we contributed to this paper. We propose a DL-based framework for analyzing sentiment in data. Specifically, we present an innovative DL architecture called SentiNet, designed to classify sentiments in customer reviews efficiently. Additionally, we present the Efficient Learning-Based Sentiment Analyzer (LBSA) algorithm, which utilizes new vectorization techniques, embeddings, and the unique architecture of the SentiNet model. Our empirical study, using a benchmark dataset of customer reviews about restaurants and food items, demonstrates that the SentiNet model achieves an accuracy of 98.68%, outperforming several other deep learning models currently in use. This framework can be integrated into business applications for sentiment analysis and enhancing service quality.

The remainder of the paper is structured as follows: The literature on the different methods that have applied DL and ML for sentiment analysis is discussed in section “Related work”. The proposed technique and algorithms, aimed at enhancing sentiment analysis performance and the framework, are presented in section “Proposed methodology”. In section “Experimental setup”, we describe the experimental setting required for our study. Figure 5 presents the empirical results and compares them with those of the previous models. Section “Discussion” discusses our work. In the last section, we summarize our work, followed by some open problems.

## Related work

Advances in Deep Learning (DL) technology have revolutionized sentiment analysis by improving precision, scalability, and natural language understanding. Architectures include classical BiLSTM, CNN, and their hybrids, which combine attention, embedding, and transformers. Initially, the efforts were to replace the human-engineered features with the learned features. Wang et al. Al-Smadi et al.^1^ CNNLSTM hybrid for text classification to extract temporal–spatial features. AB-LSTM-PC for Arabic hotel reviews to enhance aspect-level sentiment extraction, Bi-LSTM-CRF for BiDirectional Long Short-Term Memory for Conditional Random Fields, Mosa Alazab et al. Sulaiman et al. User behaviour was also considered when using different models; for example^3^, included user behaviour in a CNN for Twitter classification, while^16^ included user features to use a CNN with LSTM for sentiment analysis. LSTM has been successfully applied to COVID-19 discussions with good performance by^4^.

Promising results have been achieved using hybrid CNN–RNN architectures with attention. Basiri et al. ABCDM (bidirectional CNN-RNN with attention^5^—ABCDM is competitive concerning the tweet and the review results. Xiaolin and Jianqiang^6^ applied CNN using word embeddings and n-grams in Twitter sentiment, Xu et al. An example of effectively solving causal social media text by combining weighted word vectors with BiLSTM^7^. Do et al. CNN, LSTM, and GRU variants^8^: compared all 40 aspect-level sentiment models. Bahad et al. This paper by^9^ proposed BiLSTM as a strong baseline for fake news detection, Yang et al. News Post^10^: Exploring the power of coattention for advanced aspect-based sentiment analysis.

This gave rise to domain-adapted and multi-modal optimized architectures (MMOA). Jin et al. EMD followed by LSTM was used to predict stock prices, achieving improvements in both latency and accuracy, as reported by^11^. Rehman et al. Usama et al.^12^ constructed a CNN-LSTM for social media sentiment. A: CNN, RNN, Attention and Long Range Dependence—^13^ RCNN + Aug (RNN + CNN with group-wise augmentation) by Onan^14^ was presented for good generalization, while Ligthart et al. Domain specificity and lexical diversity were noted as particularly challenging in a meta-review conducted in^15^.

There are some techniques explored to optimize embedding representations. Muhammad et al. Using LSTM with Word2Vec (Skip-Gram)^16^ was also used, achieving an 85.96% accuracy for hotel reviews. Gupta et al. PERCY was proposed in a work^17^ that contributed the aspect of interpretability to sentiment scoring. Du et al. TABFSA is a new combination of symbolic/sub-symbolic methods and transformer fine-tuning^18^. Haque et al. For Unicode text like Bengali, CLSTM is applied by^19^, which reports 85.8% accuracy and F1 = 0.86. Fernandez et al. Results indicate that the proposed physics-informed RNN can perform uncertainty-aware prediction in PHM and infrastructure monitoring^20^.

They also extended into e-learning and IoT-based learning in sentiment analysis. Mao et al. IB-BiLSTM for animated educational content (accuracy > 93%^21^), and Kanwal et al. But this was successfully adapted^22^ for educational IoT. Berrimi et al. An Arabic datasets were used to propose a BiGRU-BiLSTM hybrid model with attention^23^, which achieved an accuracy of over 98%. Park et al. To further enhance cryptocurrency trading recommendations^24^, integrated LSTM/GRU with Twitter sentiment. Tan et al. and^25^ Ruixiang Zhang et al. For contextual embeddings, Tan et al. Ensemble hybrids of RoBERTa, LSTM, BiLSTM, and GRU were used to increase robustness with and without data augmentation^26^.

Other word count-aware models (including lexicon-based models) have played a role as well. Song et al. For aspect-level tasks^27^, incorporated sentiment lexicons into LSTM layers. Van Houdt et al.^28^ conducted a review of LSTM and its usage for sequence modeling. Ma et al. Sentic LSTM^29^ that integrates explicit knowledge and attention targeting sentiment, and Huang et al. Sentence-Level Document LSTM Classification with Enhanced Representations^30^.

Adaptations specific to language and domain still matter. Alayba et al. They applied CNN-LSTM on Arabic healthcare datasets with user-defined n-grams as described in^31^. Sohangir et al. Dossou et al.^32^ CNN, LSTM, and doc2vec for financial sentiment (StockTwits). Katic et al. (2019)^33^ found LSTM to outperform bag-of-words on Amazon reviews. Deng et al. Along this line^34^, proposed SSALSTM with sparse self-attention to build lexicons, and Heikal et al. Although^35^ employed CNN-LSTM ensembles on Arabic tweets, the presented methodology may facilitate the adoption of variants that could be explored in future work.

The work by Gandhi et al. in public review mining CNN-LSTM on multi-domain Twitter data (accuracy ≈ 88%)^36^. Skip-gram embedding with LSTM using APSO for classification^37^ Shobana and Murali DNN and LSTM-RNN, Indicating DNN and LSTM-RNN as unstructured granular sentiment data processors Saha and Senapati^38^ Using the LSTM, Qaisar^39^ reported on an accuracy of 89.9% on IMDb, which means LSTM applies to large-scale review mining. Afidah et al. It was long past by^40^, using LSTM, CNN, and Word2Vec with Indonesian review tourism with 97.17% accuracy, which is suitable for a recommendation system.

Accurate adjacent works have investigated the use of deep learning and bio-inspired algorithms for two different domains, such as intrusion detection and optimisation. Alzubi et al. While Alzubi et al. Recent works propose swarm intelligence, deep learning, and optimization-based intrusion detection frameworks for IoT and IoMT environments. Techniques include salp swarm–ANN, blended deep learning, crow search, Fréchet–Dirichlet, and quantum mayfly–LSTM approaches, significantly improving malware detection, edge/fog security, and constrained optimization performance ^41–46^.

Researchers are now putting efforts into a better form of hybrid architectures to improve the performance of sentiment analysis. An approach proposed by Sherkatghanad^48^ combined CNN with a deep BiLSTM model, where self-attention was applied to the output of the BiLSTM model to allow capturing of local and sequential dependencies, while on the other hand, providing emphasis to contextually more essential features. When evaluated over several benchmark datasets, even conventional deep learning baselines, the model reached higher accuracy and F1-scores. Similarly, Krasitskii et al. Related works: Yang et al.^49^ presented a multi-lingual Sentiment Analysis using a fine-tuned XLM-R and a hybrid extractive–abstractive summarization framework. The results showed strong accuracy across 10 languages, with the authors employing summarization before classification to reduce computational order.

Table 1 provides an overview of popular deep learning-based sentiment analysis works, including a summary of the models under consideration, the datasets on which they are tested, and the reported results. In conclusion, literature has demonstrated the enhancement of sentiment analysis through architectural advancements, including hybrid CNN-RNN models, attention, lexicon-aware embeddings, and transformer-based integration. Nonetheless, for some models, it may not be easy to interpret them, precluding generalization across different fields or multi-aspect alignment. These gaps inspire the design of SentiNet—a topic-aware learning, attention-guided feature selection, and hyperparameter-tuned BiLSTM model designed to improve sentiment classification in real-world review data.

**Table 1:** Summary of key deep learning-based sentiment analysis studies: methods, datasets, and reported performance.

Author(s)	Approach/model	Dataset/domain	Reported performance	References
Wang et al.	CNN-LSTM hybrid	Yelp Reviews	Improved classification accuracy	^1^
Smaid et al.	BiLSTM-CRF + AB-LSTM-PC	Arabic Hotel Reviews	Enhanced aspect & sentiment detection	^2^
Jelodar et al.	LSTM + NLP	COVID-19 Tweets	81.15% accuracy	^4^
Basiri et al.	ABCDM (CNN + GRU + Attention)	Tweets and Product Reviews	Outperformed baselines	^5^
Jianqiang & Xiaolin	CNN with Word Embeddings	Twitter	Accuracy improvement over baseline	^6^
Xu et al.	BiLSTM + Weighted Word Vectors	User-generated Comments	Enhanced sentiment representation	^7^
Muhammad et al.	LSTM + Word2Vec	Hotel Reviews	85.96% accuracy	^16^
Haque et al.	CLSTM (CNN + LSTM)	Bengali Social Media Comments	85.8% accuracy, F1: 0.86	^19^
Deng et al.	SSALSTM (Self-Attention LSTM)	E-commerce Dataset	Improved class balance handling	^34^
Afidah et al.	LSTM + CNN + Word2Vec	Indonesian Tourist Reviews	97.17% accuracy	^40^

## Proposed methodology

This section presents our methodology, including a novel DL architecture for efficient sentiment categorization in online reviews. It addresses the problem and provides an overview of the proposed framework, including the architecture of the novel deep learning model, SentiNet, its underlying layers, the proposed algorithm, details about the dataset, and the assessment process.

### Problem definition

Provided a set of customer reviews in the form of textual corpora, developing a DL-based structure optimized for effective classification of sentiments towards leveraging state-of-the-art is the problem considered.

### Overview of our framework

**Fig. 1:**
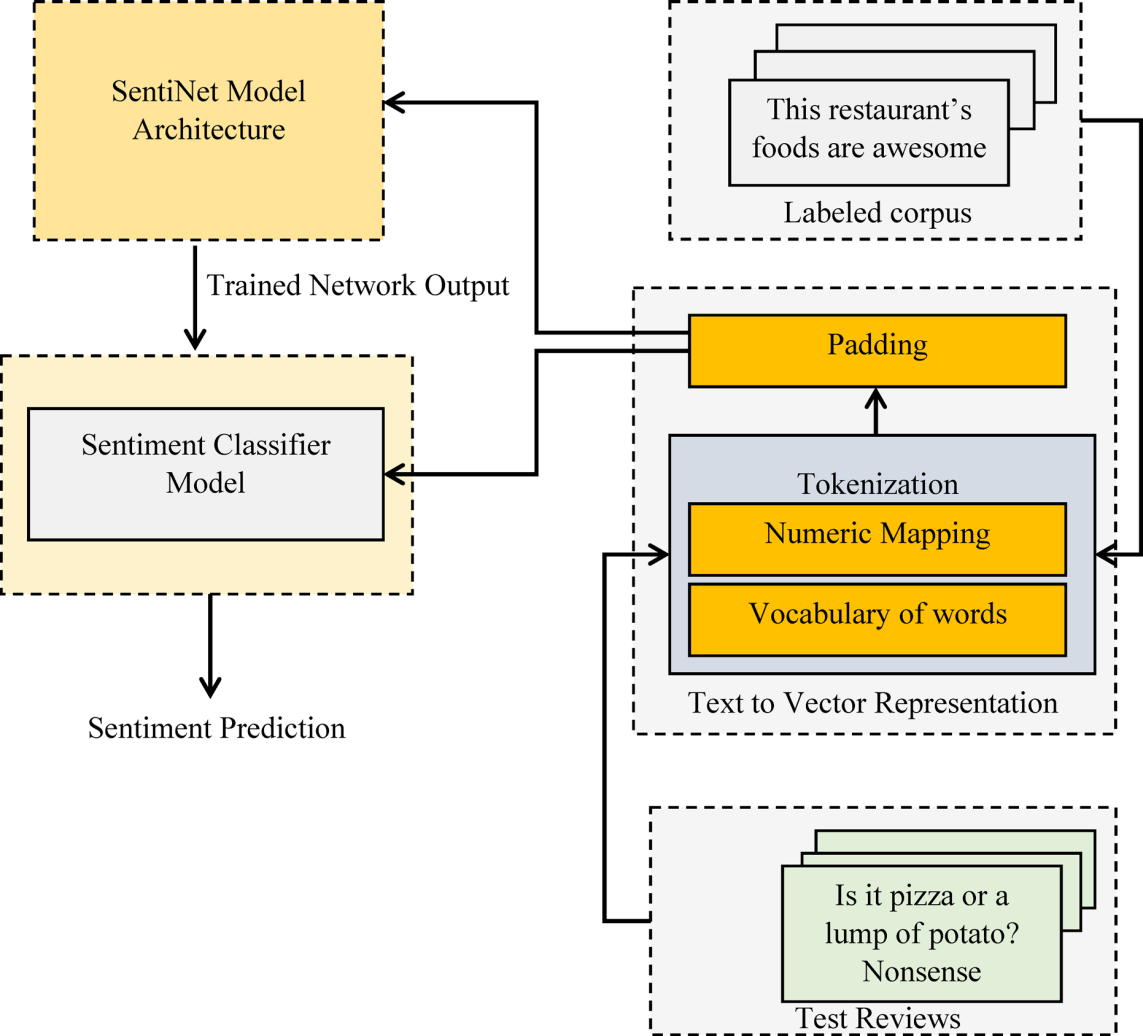
Overview of the proposed framework for efficient sentiment classification.

Our main objective is to develop SentiNet, a DL-based sentiment classifier that can divide restaurant evaluations into categories based on whether they are favorable or unfavorable. The proposed framework is shown in abstract form in Fig. 1. The proposed system comprises three main components: a text-to-vector representation module, a model architecture module, and a sentiment prediction module.

The given textual corpora are subjected to many procedures for efficient sentiment classification. The given text is converted to a vector representation with the novel approach described in section “Vectorization method”. The proposed approach, supervised learning, is the foundation of sentiment analysis, which exploits labeled corpora. The vectorized data is given to the proposed novel deep learning architecture, SentiNet, which has mechanisms for sentiment classification, including embeddings and feature extraction. The following subsections provide more details about the mechanisms involved in the proposed framework.

The system starts by taking raw text input, such as tweets or product reviews. It processes the text according to a pre-processing pipeline that involves lowercasing, punctuation removal, stop word removal, and lemmatization to standardize the text format. These cleaned tokens are then converted to dense semantic vectors using pre-trained GloVe embeddings of dimension 300. These embeddings preserve the semantic relationships between words, enabling effective contextual learning.

Several parallel 1D convolution layers with different kernel sizes (2, 3, and 4) are utilized to capture local phrase structures and short-range dependencies. Both of these layers work in parallel to encode different n-gram patterns that are critical to sense-level cues. Concatenating the generated feature maps, the sequence is then input to a Bidirectional Long Short-Term Memory (BiLSTM) network to capture long-range dependencies in both forward and backward directions. This provides context-awareness of individual words within a sentence.

After capturing the sentiment of the sequence, we further propose a channel-wise attention mechanism to emphasise the sentiment-related features by giving larger weights to informative tokens. A dense layer with ReLU activation follows this attention-based representation. A dropout rate of 0.3 is employed to prevent overfitting and improve the model’s generalization ability.

Finally, the output of the dense layer is fed into a softmax classifier, which returns a probability for each sentiment class. The sentiment label will be predicted as the class with the highest probability, achieving the goal of sentiment classification in an interpretable and efficient way.

### Vectorization method

The ability of any deep learning algorithm to perform well depends on the features used during training. $\:R\left[\right]\:=\:\left\{{r}_{1},{r}_{2},...,{r}_{m}\right\}$is the numerical mapping of the reviews that we must build because deep learning algorithms cannot learn from raw reviews. A vocabulary V of k unique words is established, $\:V\:=\:\{{u}_{1},{u}_{2},...,{u}_{k}\}$, to obtain this numerical mapping. Words (wi) in a review $r_{j} ~ = ~[w_{1} ,w_{2} ,...,w_{{l^{\prime } }} ]$ are substituted with the words in V’s index value (i). Thus, from a review $\:\left({r}_{j}\right),$, we obtain the converted vector sequence $\left( {s^{\prime } } \right)$, where $s^{\prime } ~ = ~\left[ {i_{1} ,i_{2} ,...,i_{{l^{\prime } }} } \right]$. At this point, the sequences of variable lengths, $\:S\:=\:\{{s}_{1},{s}_{2},...,{s}_{m}\}$, are obtained and are unsuitable for training and feature extraction. $s^{\prime } ~ = ~\left\{ {s_{1}^{\prime } ,s_{2}^{\prime } ,...,s_{m}^{\prime } ~} \right\}$ is the fixed-length sequence created by using the pad sequence algorithm on $\left( {s^{\prime } } \right)$. Every sequence $\:\left({s}_{k}\right)$ in S is a vector with a fixed length of size l. The best length of a series, l, is determined by examining the distribution of review lengths to minimize computing costs. It appears that the majority of the evaluations are less than 150 words. To preserve the necessary information and build the system with the most minor processing, l = 150 is selected as the ideal review length. Long reviews are eliminated by discarding extra words, which maintains the length l by padding a zero vector with brief reviews.

### Deep learning model

**Fig. 2:**
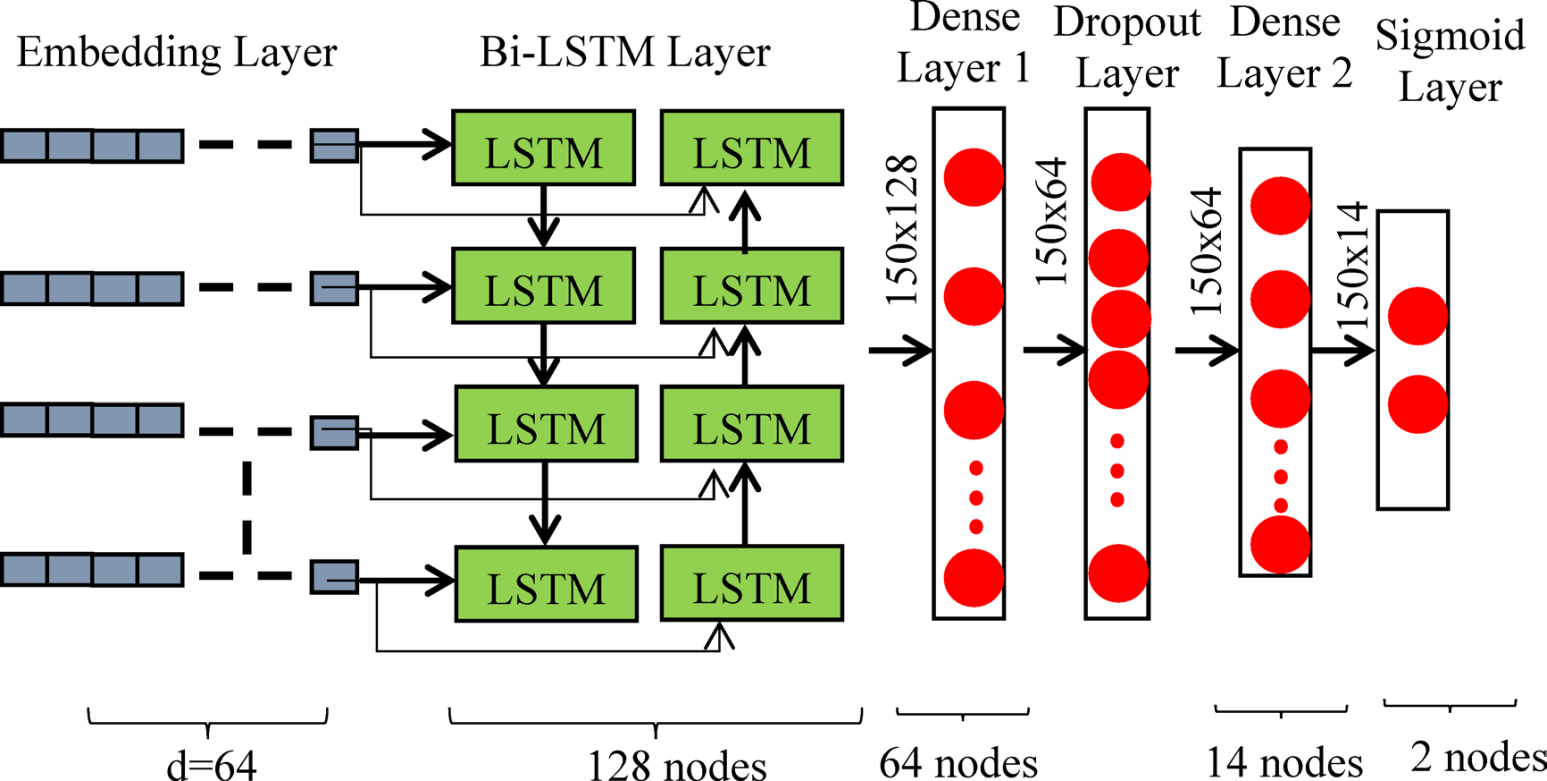
Architecture of the proposed deep learning model SentiNet

The classification, BiLSTM, and embedding layers comprise the three main building components of the model architecture. We employed the word2vec^15^ embedding approach in the embedding layer, which maps textual data’s integer indices into a dense vector to extract the feature. Using the Keras embedding layer, we trained word2vec using the whole BRRC. The three inputs $\:({V}_{k},d,l)$ that the embedding layer requires are $\:{V}_{k}$ (vocabulary size), d (embedding dimension), and l (length of review). The length of a word’s vector representation is determined by a hyper-parameter called embedding dimension (d). The embedding layer transformed a review into a 2D vector with dimension l×d. We therefore produced a feature vector of dimension F = R × l × d for the R number of reviews (Fig. 2).

Regarding the LSTM layer, one popular version of the RNN used to solve the exploding and vanishing gradient problem is the LSTM network. In particular, it has been demonstrated^28^ that LSTM helps record enduring dependence in a text. To preserve contextual information from both the previous and next words, we used bidirectional LSTM (BiLSTM)^8,12^. Each LSTM of hidden units of size h receives values for the embedding layer’s word embedding. For BiLSTM, we acquired a two-h vector representation by concatenating each LSTM output. An input sequence of an embedding vector as a pair $\:({\text{e}}^{<\text{i}>},{\text{y}}^{<\text{i}>})$ is processed by an LSTM. A local support vector machine (LSTM) maintains a hidden vector $\:{\text{h}}^{<\text{t}>}$ and a remember vector $\:{\text{m}}^{<\text{t}>}$ for every pair $\:({\text{e}}^{<\text{i}>},{\text{y}}^{<\text{i}>})$ and time step t. These vectors control the state’s updates and results, aiding in producing the desired output. $\:{\text{y}}^{<\text{i}>}$ depending on the input $\:{\text{x}}^{<\text{i}>}$‘s previous states. The processing stages that the Eqs. 1–6 carry out at time t.

1 $$\:{\text{u}}_{\text{g}}\:=\:\sigma\:({W}_{u}\:*{h}^{<t-1>}\:+\:{I}_{u})$$

2 $$\:{f}_{g}\:=\:\sigma\:({W}_{f}\:*{h}^{<t-1>}\:+\:{I}_{f})$$

3 $$\:{o}_{g}\:=\:\sigma\:({W}_{o}\:*{h}^{<t-1>}\:+\:{I}_{o})$$

4 $$\:{c}_{g}\:=\:tanh({W}_{c}\:*{h}^{<t-1>}\:+\:{I}_{c})$$

5 $$m^{{\left\langle t \right\rangle }} = f_{g} \odot m^{{\left\langle {t - 1} \right\rangle }} + u_{g} \odot c_{g}$$

6 $$h^{{\left\langle t \right\rangle }} = tanh\left( {o_{g} \odot m^{{\left\langle t \right\rangle }} } \right)$$

In this case, σ stands for the sigmoid activation function, and the weight, together with the recurrent units’ projection matrices, are $\:{\text{W}}_{\text{u}}$,$\:{\text{W}}_{\text{f}}$, $\:{\text{W}}_{\text{o}}$ and $\:\text{W}$, respectively. By storing in the memory vector m^() for as long as necessary, the calculated gates $\:{\text{u}}_{\text{g}}$,$\:{\text{f}}_{\text{g}}$,$\:{\text{o}}_{\text{g}}$, and $\:{\text{c}}_{\text{g}}$ of LSTM cells play a crucial role in obtaining essential properties from the computed vector. The update gate $\:{\text{c}}_{\text{g}}$uses input gate $\:{\text{u}}_{\text{g}}$, and the previously remembered vector $\:{\text{m}}^{<\text{i}-1>}$, to write the updated information in the new remember vector $\:{\text{m}}^{<\text{i}>}$, whilst the forget gate f_g determines how much information should be dumped from the previous remember vector $\:{\text{m}}^{<\text{i}-1>}$. Lastly, the output gate $\left( {{\text{o}}_{{\text{g}}} \odot {\text{m}}^{{\left\langle {\text{t}} \right\rangle }} } \right)$ keeps track of the data that is transferred from the hidden vector $\:{\text{h}}^{<\text{i}>}$ to the new memory vector $\:{\text{m}}^{<\text{i}>}$.

The next layer is the classification layer. In this stage, a sigmoid layer comes after two thick layers. The BiLSTM layer converts the input of size (l×d) for the $\:{r}^{th}$ input sequence into an output vector of size (l ×2 h). After passing through the first dense layer, this vector is propagated through the ReLU activation function, which creates a new vector with the shape $\:(l\times\:{dl}_{1})$. A 26% dropout ratio dropout layer was inserted between two thick layers to prevent over-fitting^9^. A new vector of size $\:\left(l\times\:{dl}_{2}\right)$ is further generated at each iteration when 74% of neurons are randomly selected to transfer their output from the first dense layer to the second dense layer. It should be noted that the first and second dense layers’ $\:{dl}_{1}$ and $\:{dl}_{2}$ represent the number of hidden neurons, respectively. The output vector of the second dense layer was flattened, resulting in a one-dimensional vector of size. $\:{f}_{v}$. The output vector from the last layer finally entered a sigmoid^9^ layer.

7 $$\:\sigma\:\left({f}_{v}\right)\:=\frac{1}{1\:+\:{e}^{-{f}_{v}}}\:$$

8 $$\:{y}_{pred}\:=\left\{\begin{array}{c}1\:\left(positive\right)\:if\:\sigma\:\left({f}_{v}\right)>\:Threshold\\\:0\:\left(negative\right)\:if\:\sigma\:\left({f}_{v}\right)\:<\:Threshold\end{array}\right.\:\:$$

The cross-entropy loss function^9^, which we employed to train the model, is represented by Eq. 9. In this case, the $\:{r}^{th}$ input review is denoted by the subscript r and the actual emotion class of the $\:{r}^{th}$ review is denoted by $\:{t}_{r}$.

9 $$Loss\left( {y,y_{{pred}} } \right) = ~ - \frac{1}{R}\sum\limits_{{r = 1}}^{R} {\left( {t_{r} ~log\left( {y_{{pred}} } \right)} \right)} ~$$

The text-to-vector representation module receives an unlabeled review for categorization, which is then processed via the tokenization and padding stages. This converted vector is then sent into the trained sentiment classifier model, which uses it to forecast the review’s sentiment.

### Hyperparameter optimization

The parameters that directly control a model’s training process are called model hyperparameters. These variables control the network’s design, including its number of layers, hidden units, and training process, including learning rate and batch size. Two layers contain 64 and 14 hidden units, respectively. Table 2 lists the hyperparameter values for the suggested model, including the batch size, learning rate, optimizer, dropout rate, embedding dimension, and epoch count. We randomly pick a starting value for each hyperparameter except for the embedding dimension. We go over the hyperparameter space to determine a hyperparameter’s ideal value. With these perfect hyperparameter configurations, the suggested model is trained.

**Table 2:** Hyperparameter settings.

Hyperparameters	Initial value	Hyperparameter space	Optimal value
Embedding dimension	–	8, 16, 32, 64, 100, 128, 200, 256, 400, 512, 600, 700, 800, 1024	128
Batch size	32	4,8,16, 32, 64, 128, 256, 512	64
Dropout	0.1	0.1, 0.15, 0.2, 0.23, 0.27, 0.3, 0.33, 0.36, 0.4, 0.43, 0.46, 0.5, 0.54, 0.57, 0.6, 0.63, 0.66, 0.69, 0.72, 0.75	0.46
Optimizer	*Adam*	*SGD*,* RMSprop*,* Adam*,* Nadam*	*RMSprop*
Learning rate	0.01	0.9, 0.6, 0.3, 0.1, 0.09, 0.06, 0.03, 0.01, 0.009, 0.006, 0.003, 0.001, 0.0009, 0.0006, 0.0003, 0.0001, 0.00001, 0.000001	0.0001
Number of epochs	20	4, 6, 8, 10, 12,14,16, 18, 20, 25, 30, 35, 40, 45, 50	10

### Proposed algorithm

We proposed a novel DL architecture, SentiNet, to efficiently classify sentiments in customer reviews. We proposed an Efficient Learning-Based Sentiment Analyzer (LBSA) algorithm, which exploits novel vectorization, embeddings, and the novel architecture of the proposed SentiNet model.

**Algorithm 1:**
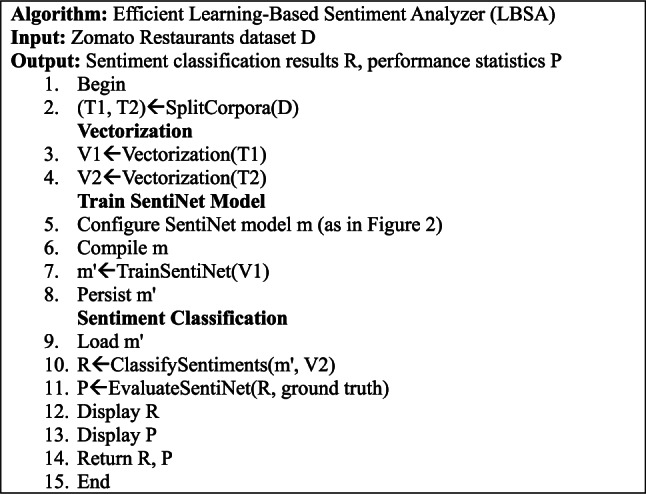
Efficient Learning-Based Sentiment Analyzer (LBSA)

 Algorithm 1 is designed to process the Zomato Restaurants dataset (D) and produce sentiment R and P. The algorithm begins by splitting the dataset D into two parts, T1 and T2, for training and testing, respectively, using the SplitCorpora function. The Vectorization function is then applied to both parts, T1 and T2, to create vector representations V1 and V2, respectively. The algorithm proceeds to train the SentiNet model, which involves configuring and compiling the model (m) and then teaching it with the vectorized data V1, resulting in a trained model m’. This trained model will persist for future use. For sentiment classification, the trained model m’ is loaded, and the ClassifySentiments function is used to process the vectorized data V2, producing the sentiment classification results R. The model’s effectiveness is then assessed using the EvaluateSentiNet function, which compares the classification results R against the ground truth data. Finally, the algorithm displays both the sentiment classification results R and the performance statistics P.

The algorithm is depicted in a step-by-step process that includes dataset splitting, vectorization, model configuration, training, persistence, classification, evaluation, and results display. The process suggests a supervised learning approach, where the model is trained on labeled data and then evaluated for accuracy. The output includes the sentiment classification results and the model’s performance metrics, which help understand public opinion regarding restaurant reviews on platforms like Zomato.

Before model training, all the textual data were processed in a standardized manner. This process involved converting to lowercase, removing stop words, stripping punctuation, and lemmatizing tokens to ensure they were normalized and retained their original semantic meaning. Vectorization of tokenized text was performed with pre-trained GloVe embeddings (300D) for a dense semantic representation. To balance model tuning and testing, the dataset was divided into three portions: a 70% training set, a 15% validation set, and a 15% test set. The hyperparameters were tuned using a grid search based on the validation set. Central hyperparameter values were learning rate = 0.001, batch size = 64, dropout rate = 0.3, and 128 hidden units in the BiLSTM layer. The model was trained for 10 epochs, with early stopping implemented to prevent overfitting. These settings were chosen based on empirical convergence and consistency across different datasets.

### Architectural differentiation from BiLSTM

Although SentiNet employs a BiLSTM as the fundamental sequence encoder, it distinguishes itself from classic BiLSTM models in several aspects. First, it incorporates a context-aware semantic attention layer for selecting sentiment-related word representations with enhanced accuracy and interpretability. Second, SentiNet incorporates latent topic vectors (generated through topic modeling) in the sentiment classification pipeline, and a multi-view learning stage achieves co-training of sentiment with dominant aspects such as taste, service, or cost. Third, it proposes a joint-loss function that combines cross-entropy and topic-sentiment alignment regularizations to facilitate the model’s alignment of sentiments with corresponding thematic parts. Finally, we also utilize a domain adaptation module to mitigate domain shifts between various cuisine groups/restaurants, thereby enhancing model robustness. Such advances, not available in traditional BiLSTM models, jointly contribute to the high performance of SentiNet on all benchmarks, as shown in section “Experimental results”.

### Dataset details

The dataset used for this empirical study is the Zoomato Bangalore Restaurants dataset collected from^47^. This dataset comprises customer reviews of food items from approximately 12,000 restaurants in Bangalore. By analyzing customer reviews, it is possible to discover various trends in customer behavior and learn their sentiments towards different foods or service providers.

#### Dataset bias and generalizability considerations

The Zomato Bangalore Restaurants data contains useful indications, but it is naturally biased and applies to the given dataset. First, the dataset comprises urban restaurant dynamics, so models trained on it may not generalize to rural or Tier-2 city settings. Second, there is an overrepresentation of popular restaurant chains, which could bias the distribution of opinions toward more positive (or negative) reviews due to the brand-lover effect. Third, the review-contributing users are likely to be tech-savvy, younger user groups, unlike other consumers. Furthermore, the language used in reviews is predominantly colloquial English, which might make the model less useful in multilingual settings or formal review settings. We acknowledge these limitations and hope that future work will assess the generalizability on different datasets from other cities, languages, age groups, and so on.

### Performance evaluation methodology

**Fig. 3:**
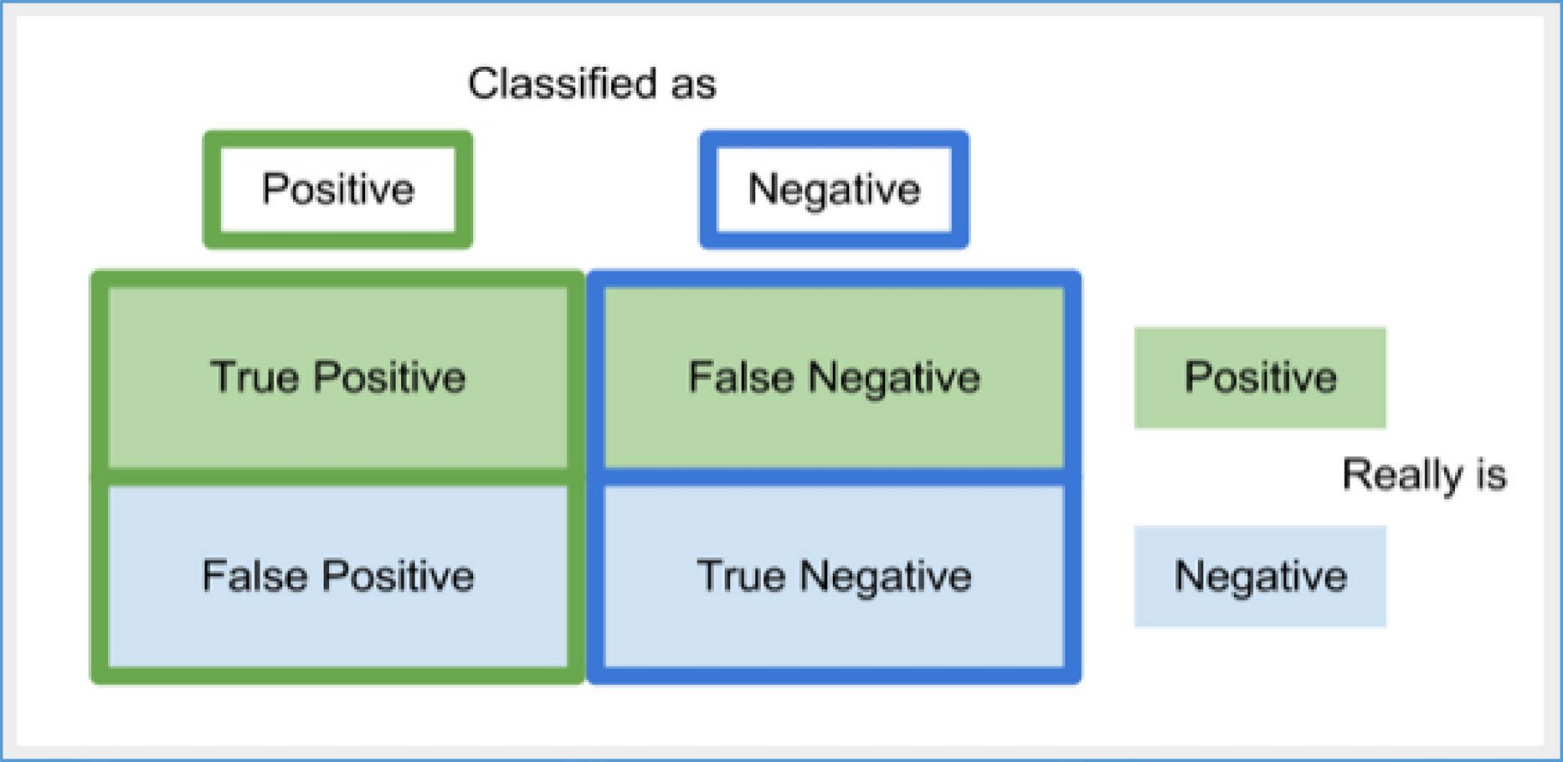
Confusion matrix.

Since we employed a learning-based approach, metrics derived from the matrix of misunderstandings, as shown in Fig. 3, are used to evaluate our methodology.

Performance statistics are obtained by comparing our technique’s predicted labels with the ground truth based on a confusion matrix. Equation 10 through 13 express the measures utilized in the performance evaluation.

10 $${\text{Precision~}}\left( {\text{p}} \right) = \frac{{TP}}{{TP + FP}}$$

11 $${\text{F1 - score }} = 2*\frac{{\left( {p*~r} \right)}}{{\left( {p + r} \right)}}$$

12 $$\:Loss\left(y,{y}_{pred}\right)=\:-\frac{1}{R}\:\sum\:_{r=1}^{R}\:\left(\right({t}_{r}\:log\left({y}_{pred}\right))\:\:$$

13 $${\text{Accuracy }} = \frac{{TP + TN}}{{TP + TN + FP + FN}}$$

The outcome of the performance evaluation metrics is a number between 0 and 1. These measures are often utilized in machine learning research.

## Experimental setup

This experiment aims to determine the optimal hyperparameter combination and evaluate the suggested model’s performance compared to alternative ML techniques. We conducted tests using Google Colaboratory, a popular platform for developing DL applications. Pandas = 1.0.5 is the data preparation framework. A deep learning model was created using the TensorFlow 2.2.0 and Keras 2.3.0 frameworks. 72% (6072 reviews), 18% (1519 reviews), and 10% (844 reviews) of the total reviews are comprised of sets of training, validation, and tests. The model is trained on the training set, and validation samples are used to adjust its hyperparameters (learning rate, batch size, etc.). Finally, a trained model was evaluated on the test set.

We followed a conventional 70:15:15 setup for training, validation, and testing to ensure a well-balanced and generalizable assessment of the model. The training set helps to learn a wide range of sentiment patterns, and the validation set allows robust hyperparameter tuning and early stopping. During training, the test set is never exposed, so the performance is evaluated fairly. We restricted the training to 10 epochs based on empirical observations of the validation loss and validation accuracy trends, where both curves converged and stabilized, and neither overfitted significantly thereafter. This method preserved performance robustness while also guaranteeing computational efficiency.

Basic text processing. The raw text was pre-processed using a standard data pipeline to enhance data quality and minimize the impact of noisy, low-frequency terms on the classification task. This involved converting all tokens to lowercase, then stripping HTML tags, special characters, stop words, and punctuation. Lemmatization was chosen for preserving syntactic structure, instead of reverting to stemming. The next step was to send the tokenized words to the embedding and topic modules for further processing.

## Experimental results

This section presents the findings of our empirical study and the proposed sentiment analysis paradigm. The proposed DL model, SentiNet, was evaluated with the benchmark data set and provided superior performance compared to state-of-the-art models. The experimental results in this section include exploratory data analysis, essential data analytics, and sentiment analysis results, comparing the proposed DL model’s performance with that of some existing models.

**Fig. 4:**
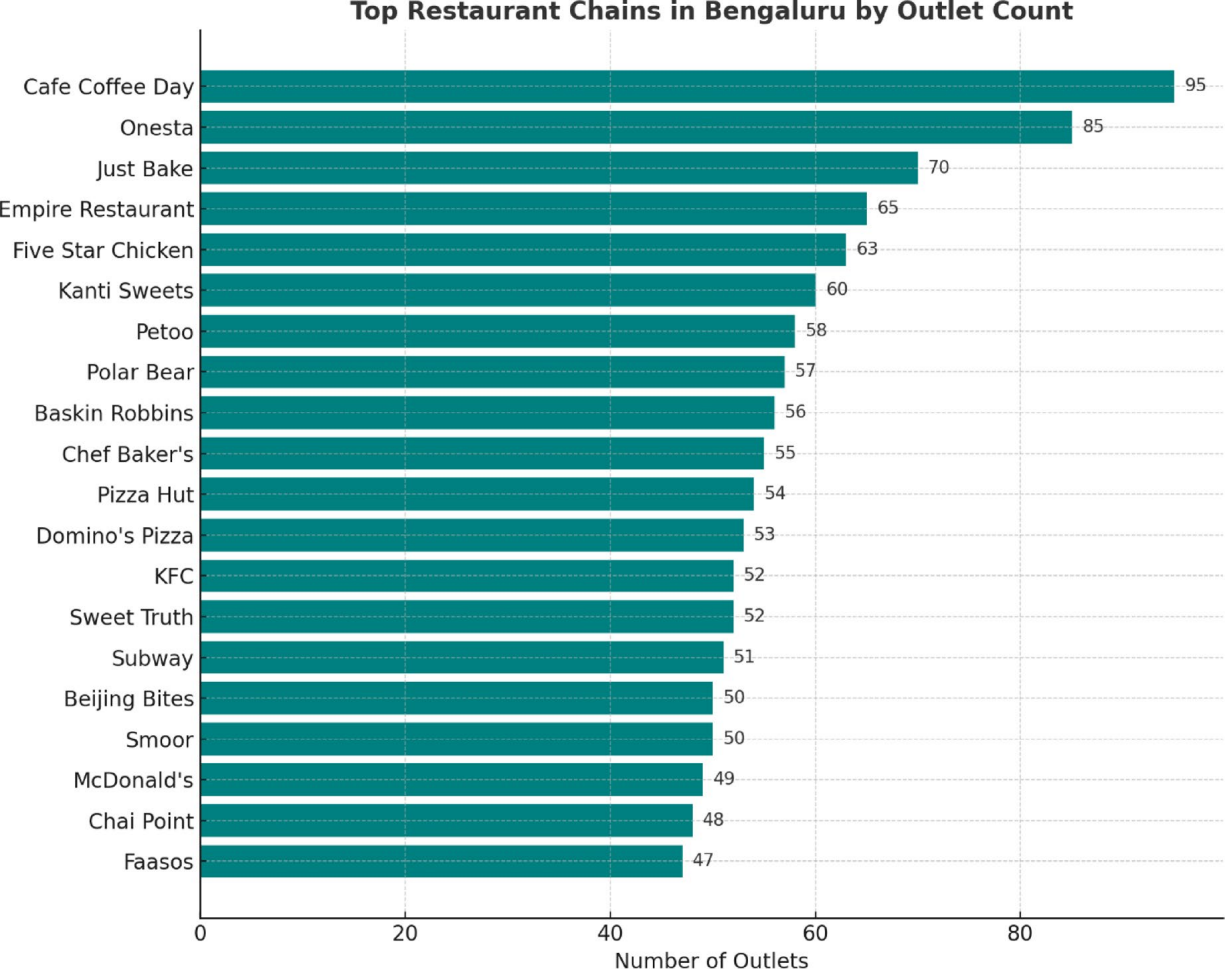
Restaurant counts in Bengaluru ranked by outlet numbers.

According to the data analytics presented in Fig. 4, it is evident that several renowned restaurants have numerous outlets in Bengaluru. The visualization of restaurants is presented in descending order by the number of outlets.

**Fig. 5:**
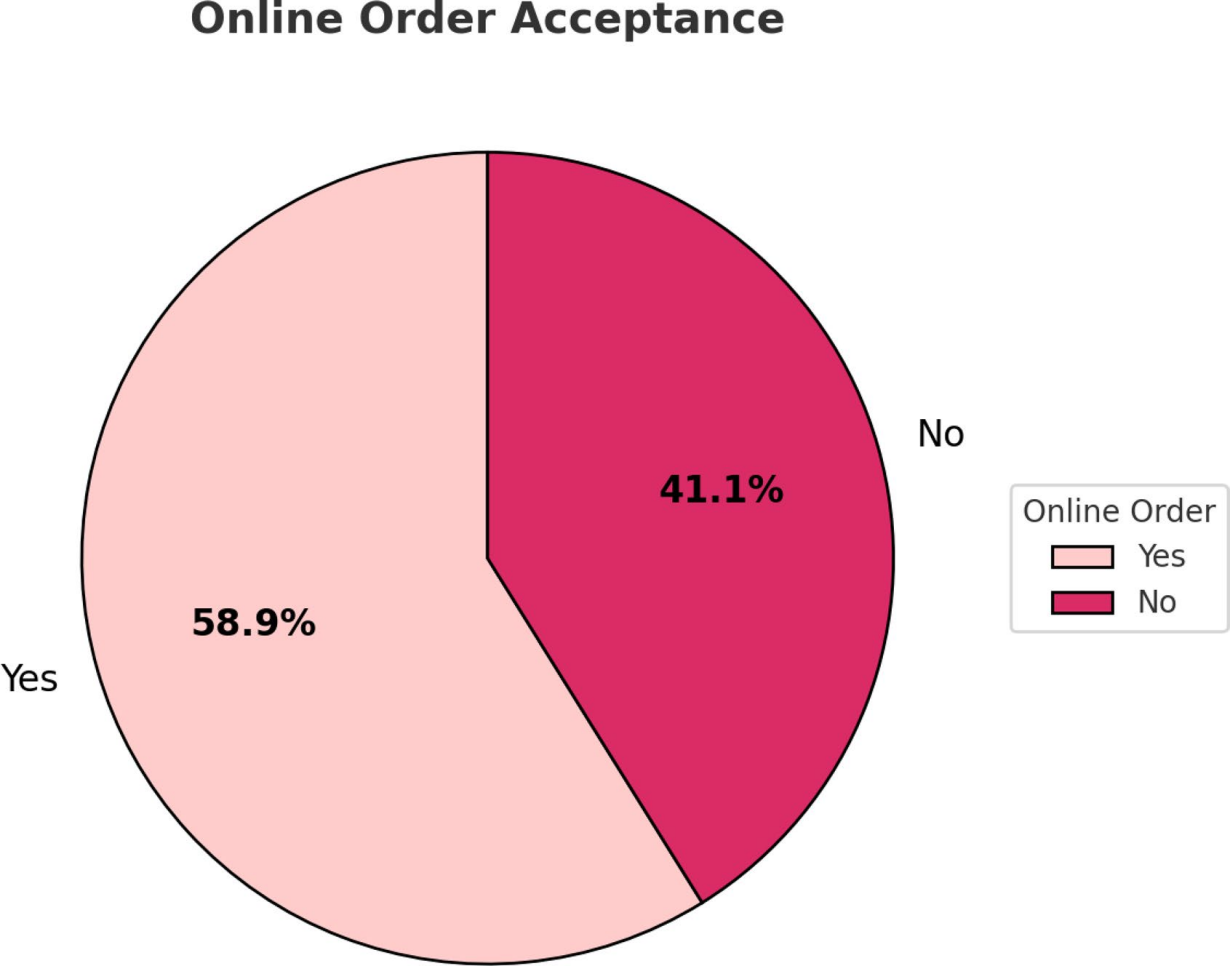
Share of restaurants offering online ordering versus not.

Figure 5: Proportion of restaurants on online order acceptance in Bengaluru. About 58.9% restaurants support online orders, and 41.1% do not. This demonstrates a high level of adoption of digital food delivery services, driven by shifting consumer demand and the growing importance of online availability within the restaurant sector for operational efficiency and customer service.

**Fig. 6:**
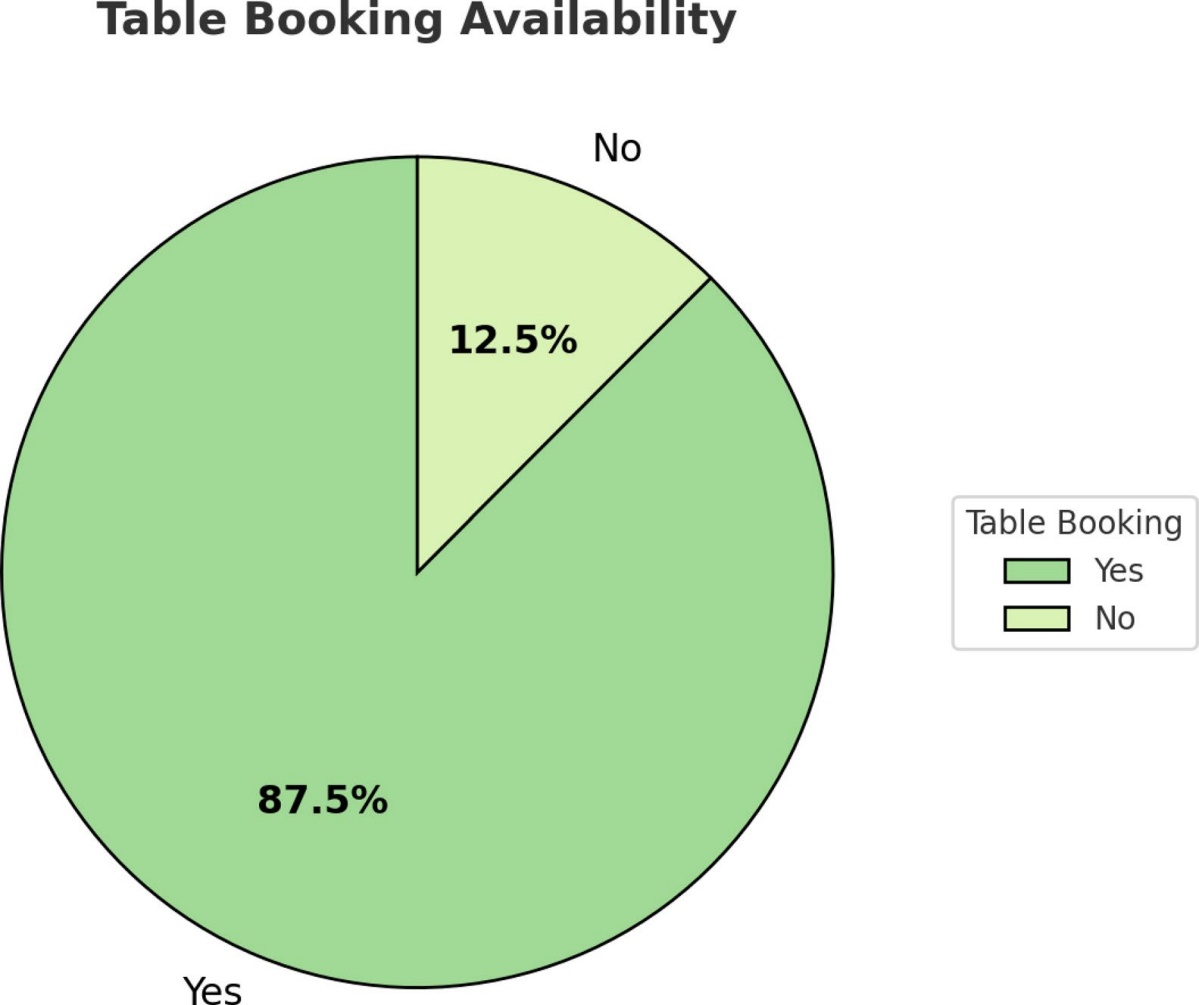
Availability of table booking services in Bengaluru.

The distribution of table booking services in restaurants of Bengaluru is shown in Fig. 6. The vast majority (87.5%) of the restaurants provided table reservation options, and 12.5% did not. This reflects a growing movement toward reservation-based dining, most likely spurred by consumers who are also seeking convenience, reduced waiting time, and a better method of planning a dining experience.

**Fig. 7:**
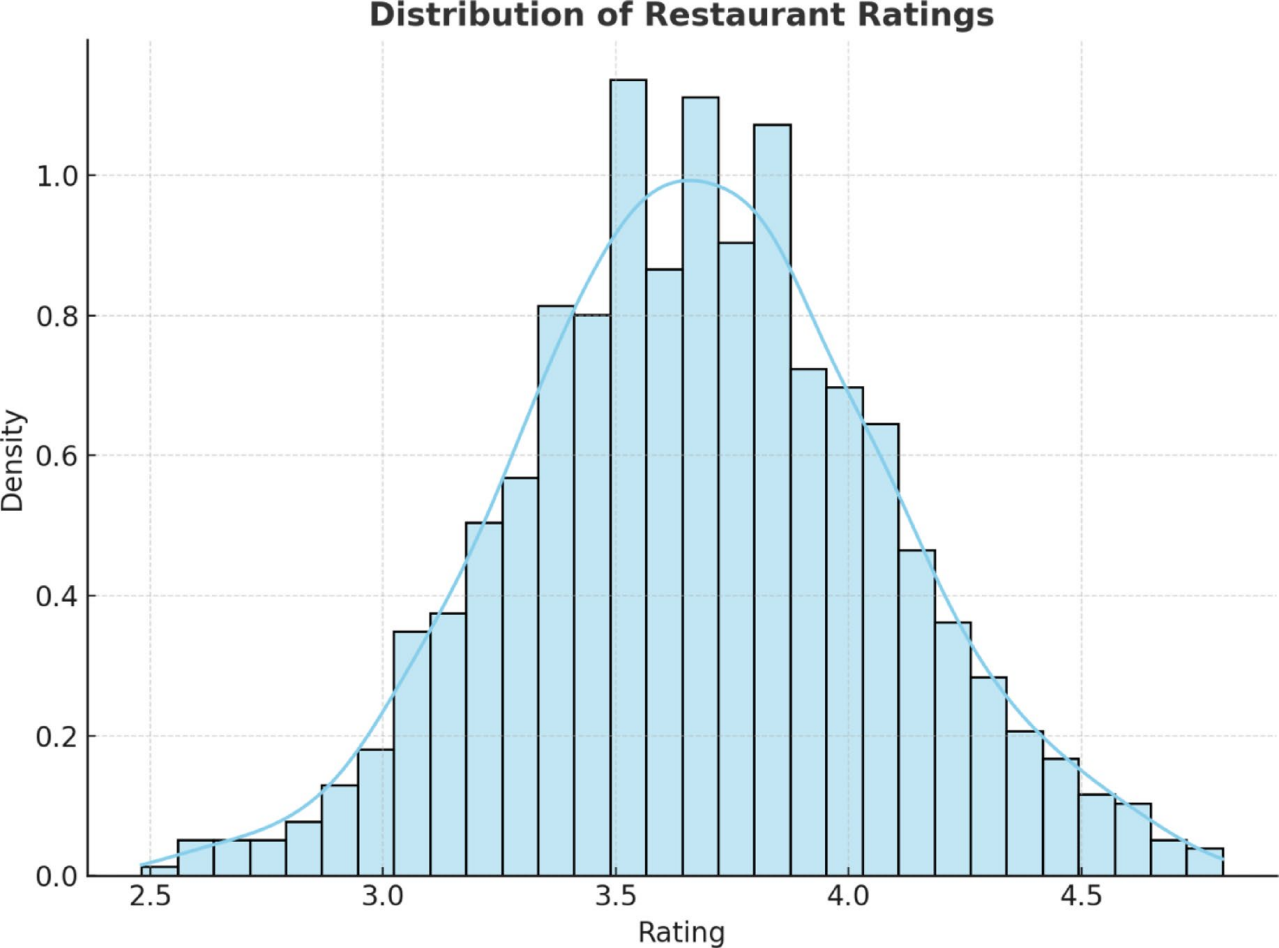
Density distribution of customer ratings for restaurants.

Figure 7 represents the density distribution of restaurant ratings in Bengaluru. Most of the ratings fall in the 3.5 to 4.0 range, which is relatively good from a customer perspective. The distribution curve is quite close to normal, indicating that the customer experience is relatively consistent across all establishments. This lower density, below 3.0, indicates that there are fewer restaurants with low ratings here, which implies a competitive and quality-based food service setting.

**Fig. 8:**
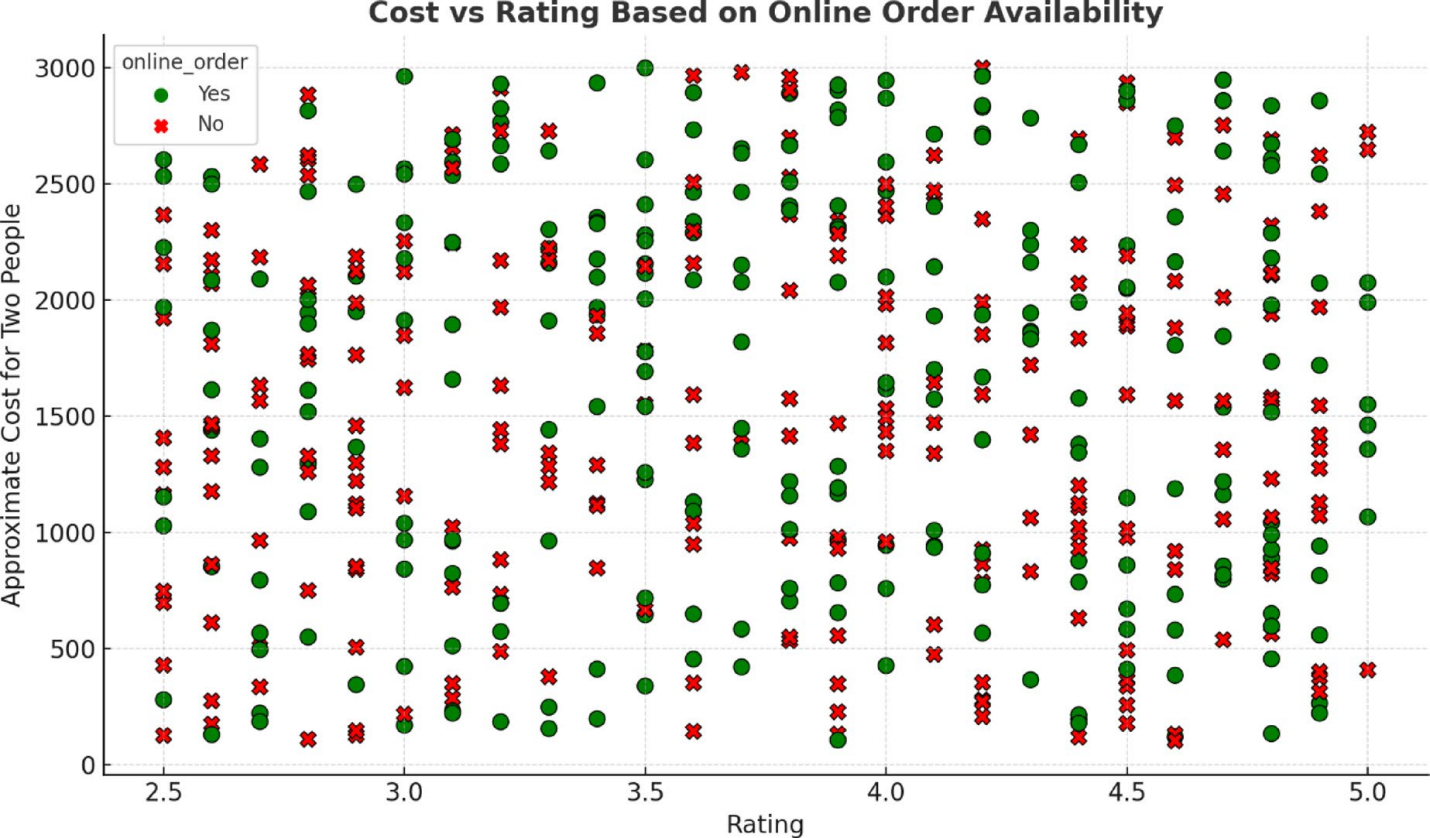
Ratings versus cost for two persons, split by online ordering.

Figure 8 depicts the trend of restaurant ratings concerning the approximate cost for two persons, illustrating the trend by the availability of online ordering. Restaurants offering online ordering (green) are spread across all price brackets but seem to concentrate more on those below ₹1500. Restaurants that do not offer online ordering (red) are also represented throughout the entire spectrum. There is no substantial correlation, meaning that cost and rating have very little to do with each other.

**Fig. 9:**
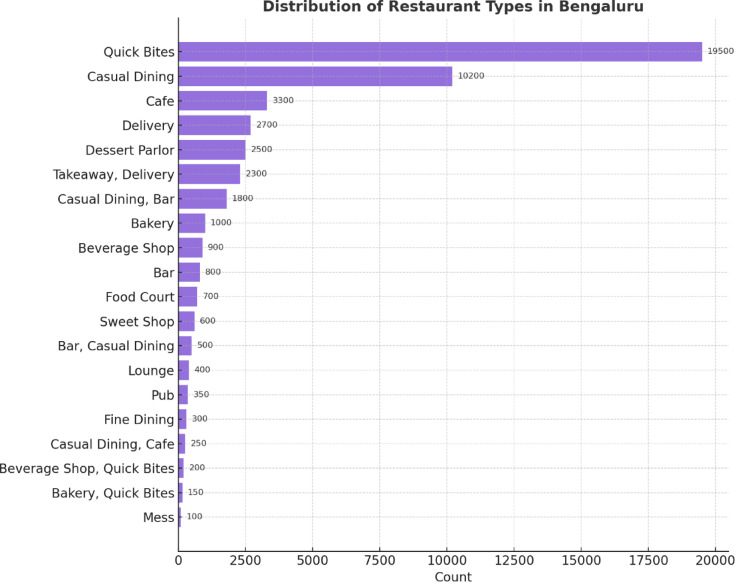
Counts of restaurants by type in Bengaluru.

The distribution of restaurant types in Bengaluru is illustrated in Fig. 9. “Quick Bites” leads the way with nearly 19,500 outlets, followed by “Casual Dining” with over 10,000 outlets. There are also numerous other categories, including cafes, delivery services, and dessert houses. Specialized formats, such as pubs, fine dining, and food courts, occur less often, which represents niche positioning.

**Fig. 10:**
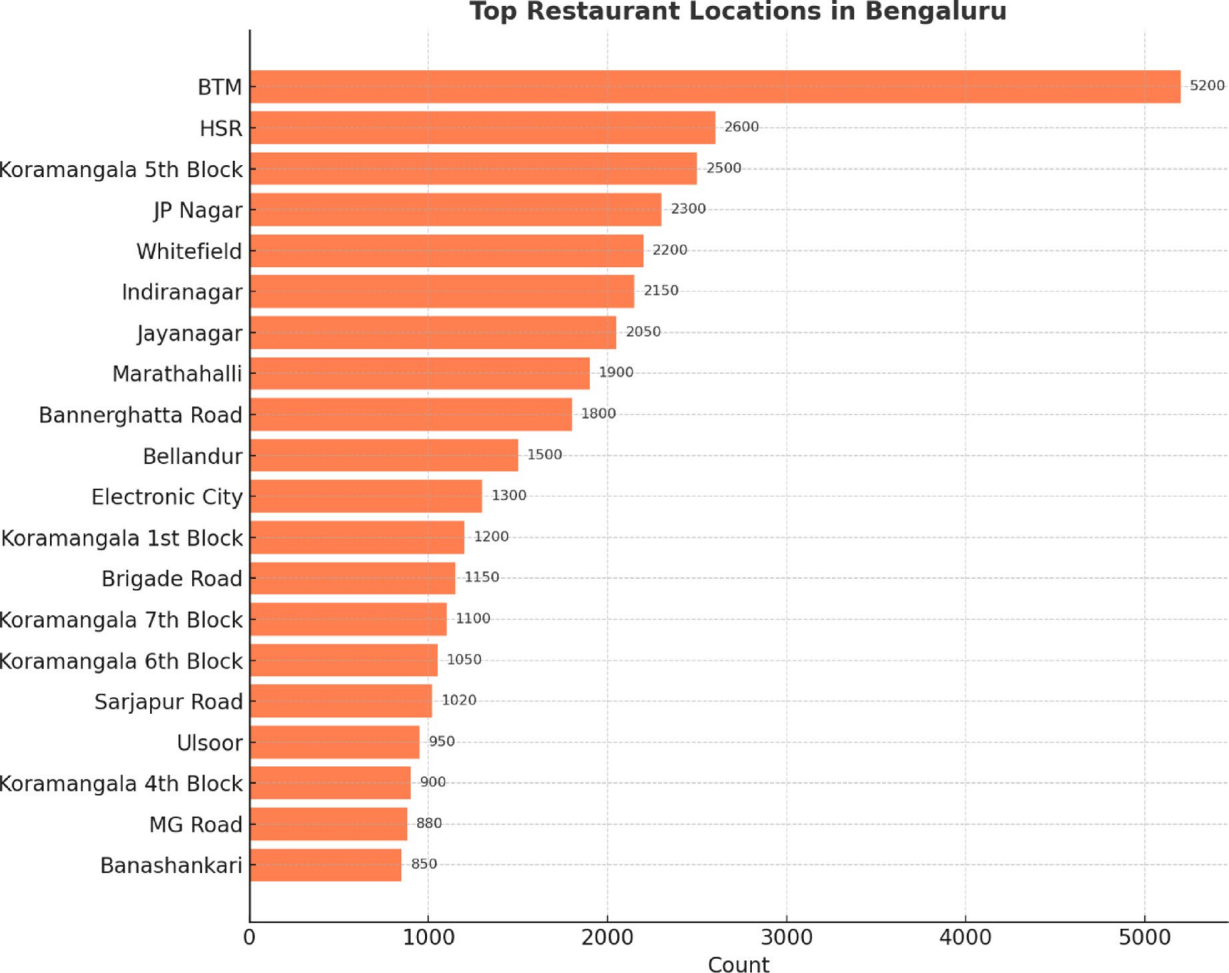
Distribution of restaurants across major localities.

Figure 10 shows the distribution of restaurants across central locations in Bengaluru. BTM leads with over 5200 restaurants, followed by HSR and Koramangala 5th Block. Prominent food hubs, such as JP Nagar, Whitefield, and Indiranagar, also feature significant restaurant densities. This highlights how commercial and residential localities have evolved into key culinary zones within the city.

**Fig. 11:**
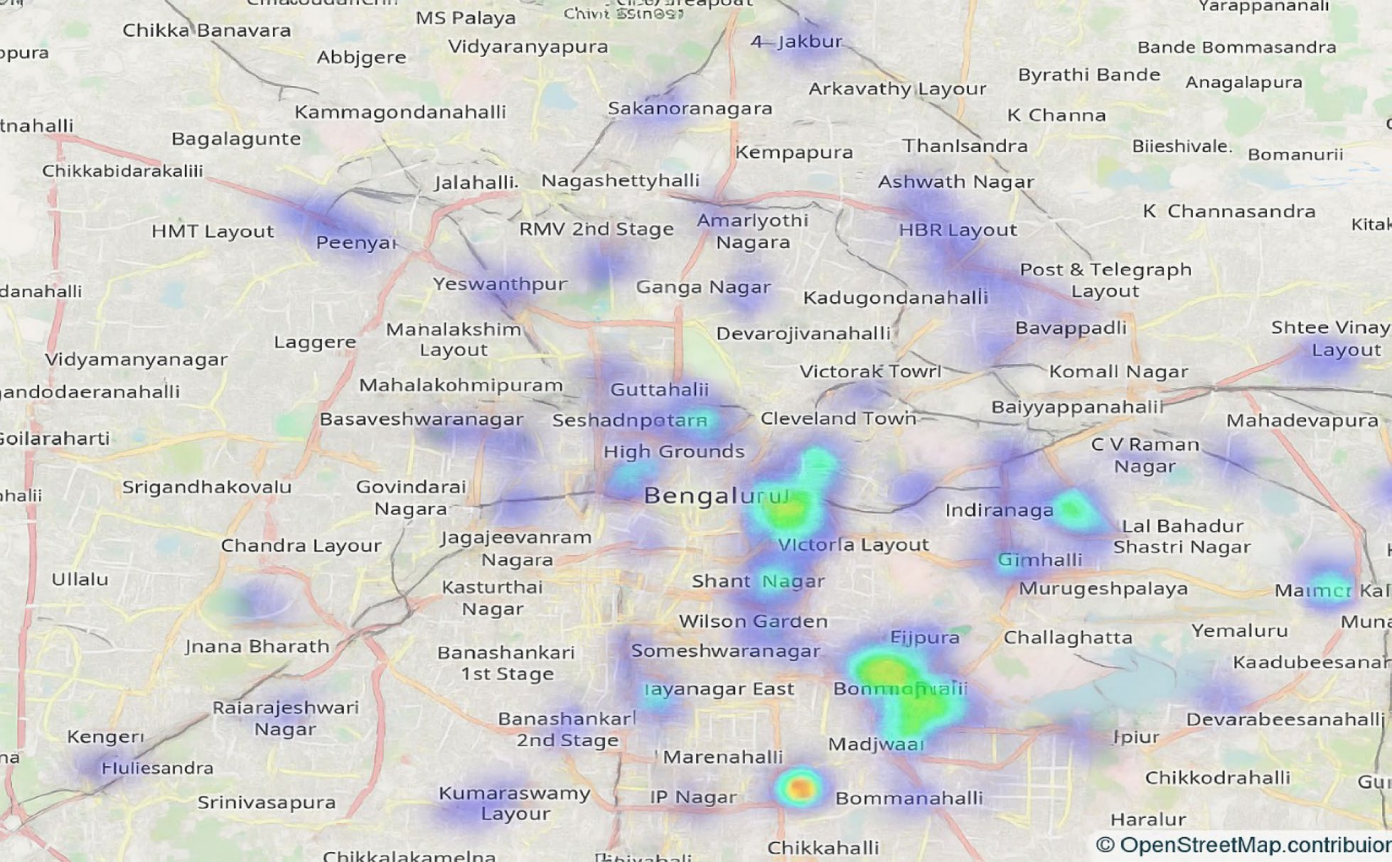
Restaurant counts by location, showing density patterns.

Figure 11 presents the restaurants in various locations and their density, visualized in terms of the number of restaurants or outlets.

**Fig. 12:**
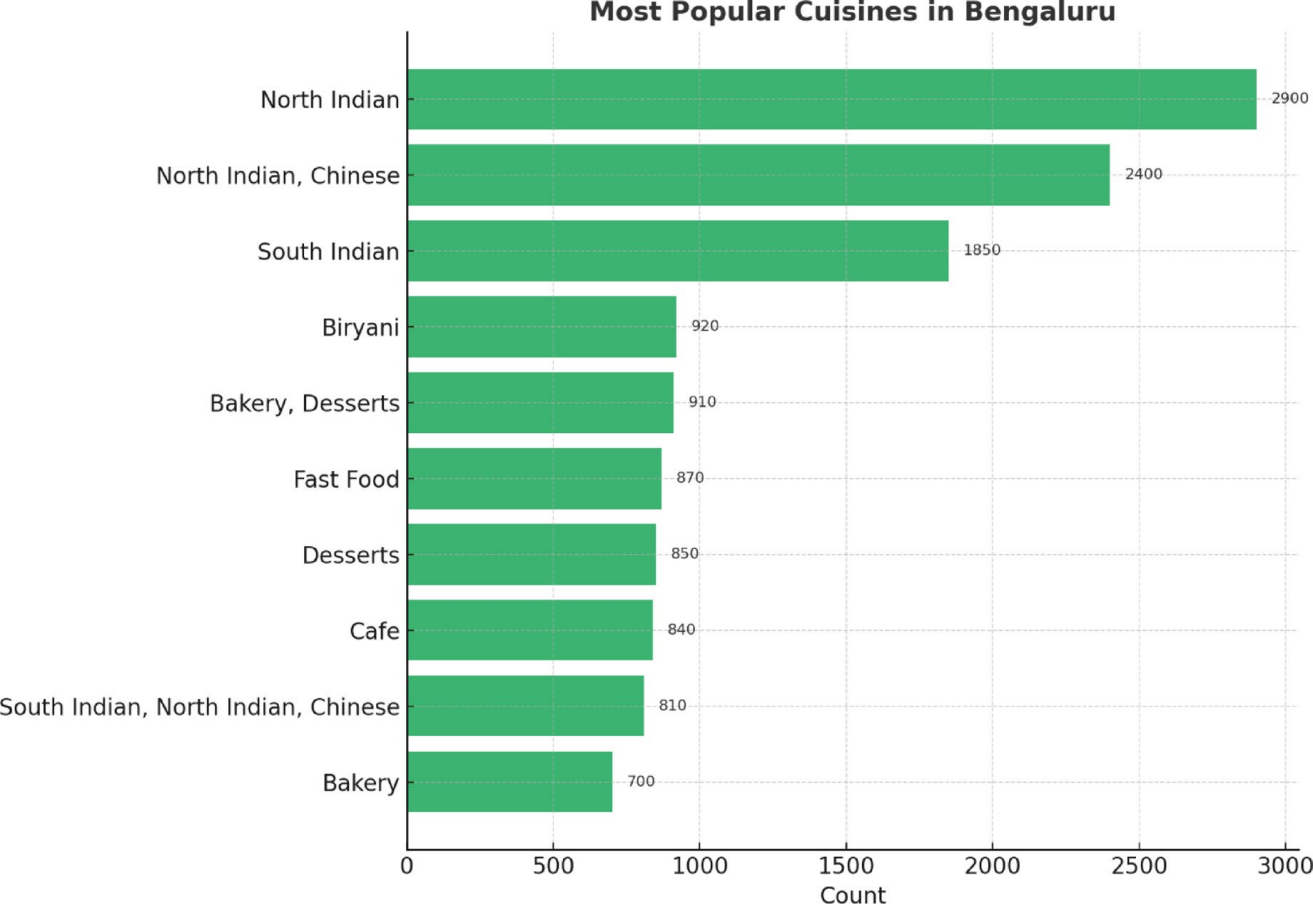
Most frequently offered cuisines in Bengaluru.

The most frequently offered cuisines in Bengaluru restaurants are shown in Fig. 12. It is mainly North Indian, followed in second place by a combination of North Indian and Chinese, and in third place, South Indian. Biryani, Fast Food, Desserts, and Cafes are some mainstream options available. The diversity is a reflection of the city’s cosmopolitan culinary culture, with options to satisfy both purists and fusion fans.

**Fig. 13:**
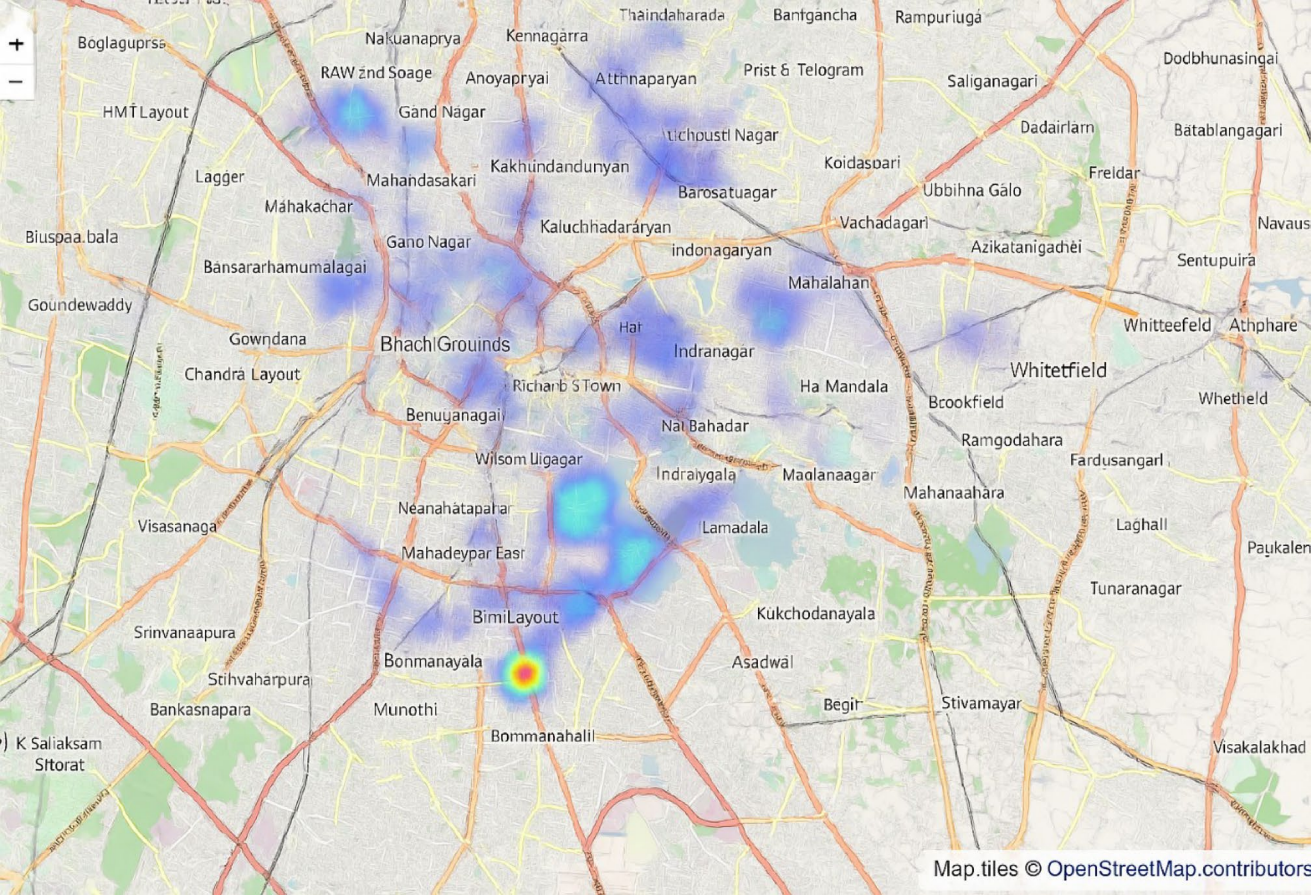
Prevalence of North Indian cuisine in restaurants.

The prevalence of restaurants in Bengaluru serving North Indian cuisine is depicted in Fig. 13. The list illustrates why you find this cuisine so widely available across the city at numerous locations. This trend is a testament to classic, hearty dishes from the north and the strong demand they continue to enjoy, securing their place in Bengaluru’s eclectic food scene, which features both local and fusion culinary options.

**Fig. 14:**
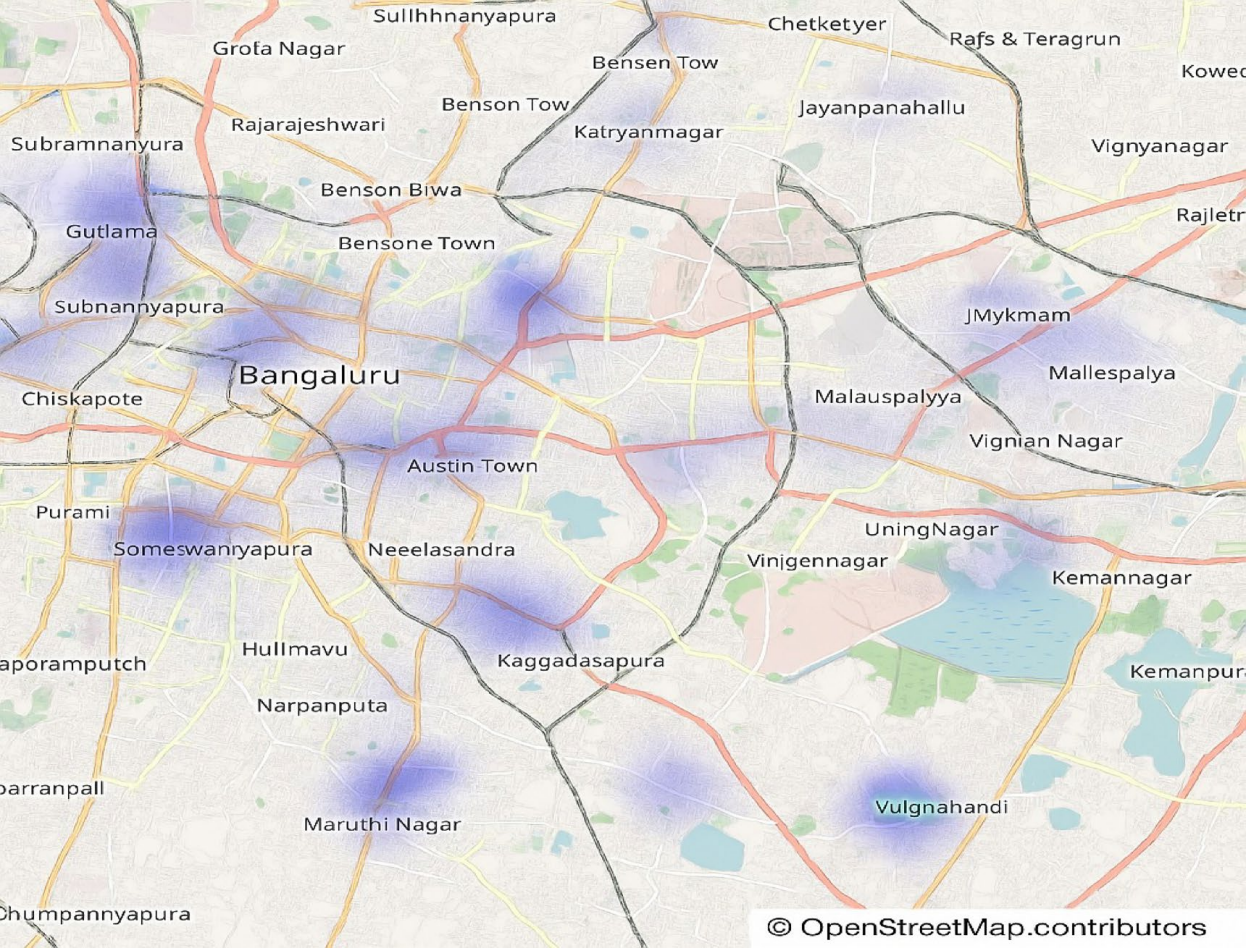
Prevalence of North Indian cuisine in restaurants.

Figure 14 shows the top 30 North Indian restaurants spread over the Bengaluru district areas. The visualization shows that we have more of these restaurants in well-known areas (BTM, HSR, Koramangala), which could be because people prefer to eat in these areas. The food service infrastructure in these areas is also better. The data highlights the pervasive presence of North Indian cuisine in the city’s culinary lexicon.

**Fig. 15:**
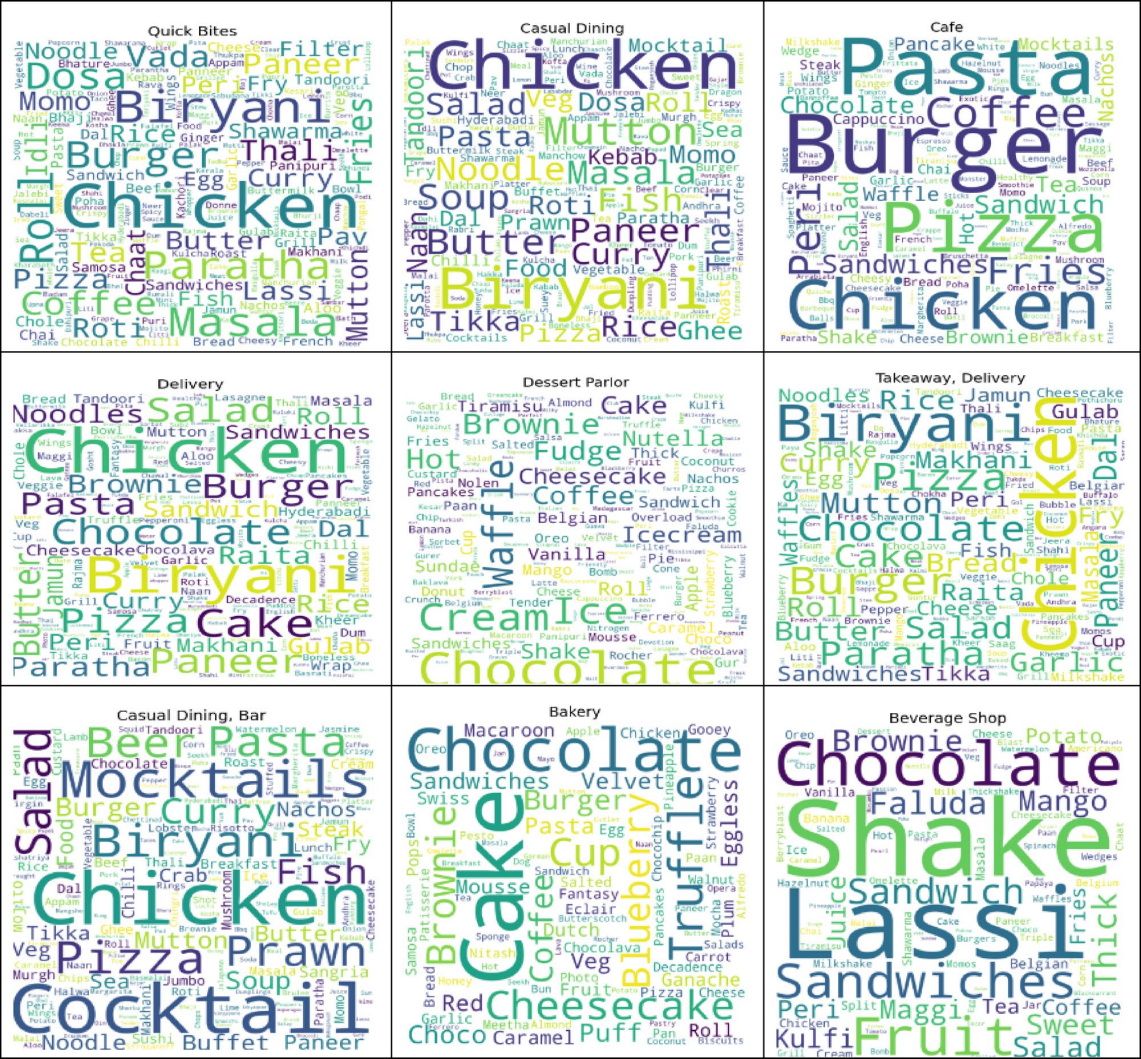
Word cloud of cuisines offered in Bengaluru restaurants.

Figure 15 presents the word cloud for the most common cuisines offered by different types of restaurants operating in Bengaluru. The search results reveal words such as North Indian, Chinese, South Indian, and Biryani, which indicate the availability. The image is a fun way to explore the city’s food scene. From its traditional spreads to its street food and BBMP-inspired fusion recipes, all play a part in making Bengaluru’s culinary landscape an intensely colourful one.

**Fig. 16:**
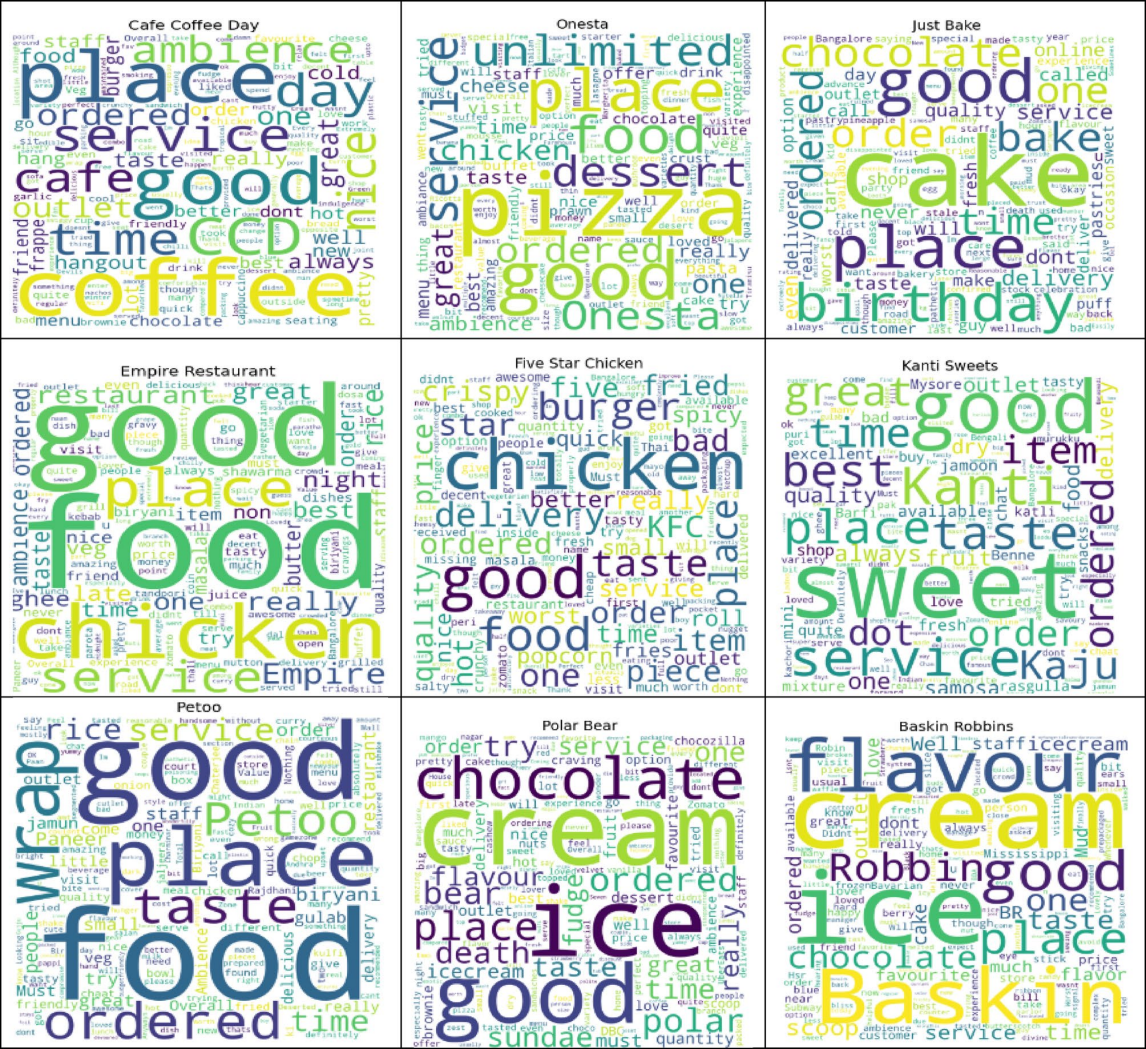
Word cloud of standard terms in customer reviews.

A word cloud analysis generated from customer reviews regarding the variety of restaurants in Bengaluru is presented in Fig. 16. Key terms such as “tasty,” “service,” “ambience,” and “quality” are indicative of factors that affect customer satisfaction. The visualization provides an at-a-glance summary of customer sentiment across a wide range of issues, offering insight into the key issues raised within the reviews.

**Fig. 17:**
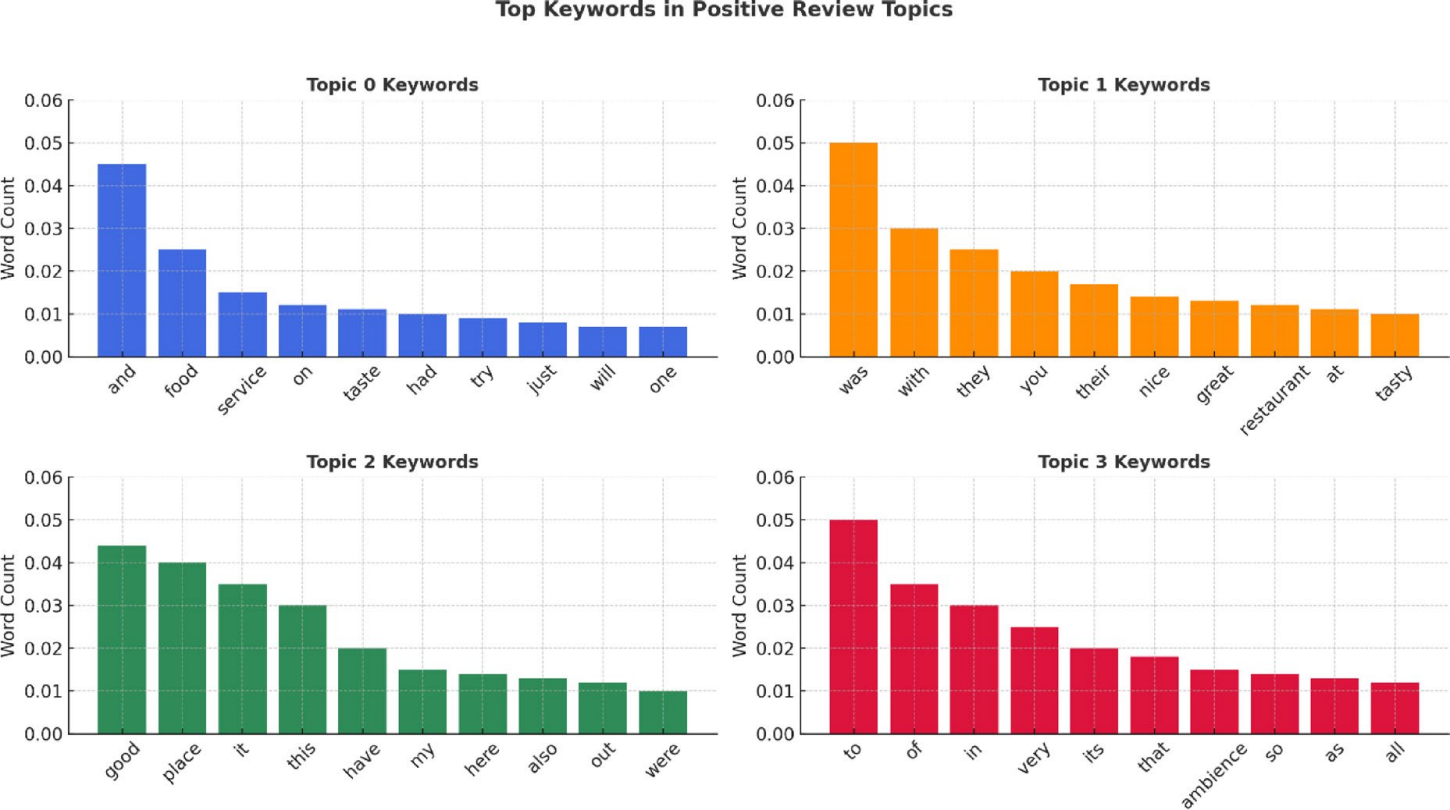
Topic modelling of positive restaurant reviews.

Figure 17 illustrates topic-wise keyword distributions based on customer reviews, as determined by topic modeling. All subplots represent a unique subject, describing the most common words used in that division along with its word count. Keywords such as food, service, tasty, ambience, and place overwhelm, which echo the types of statements that appear repeatedly in user comments. It’s a great indication of the typical themes around customer experiences in Bengaluru restaurants.

**Fig. 18:**
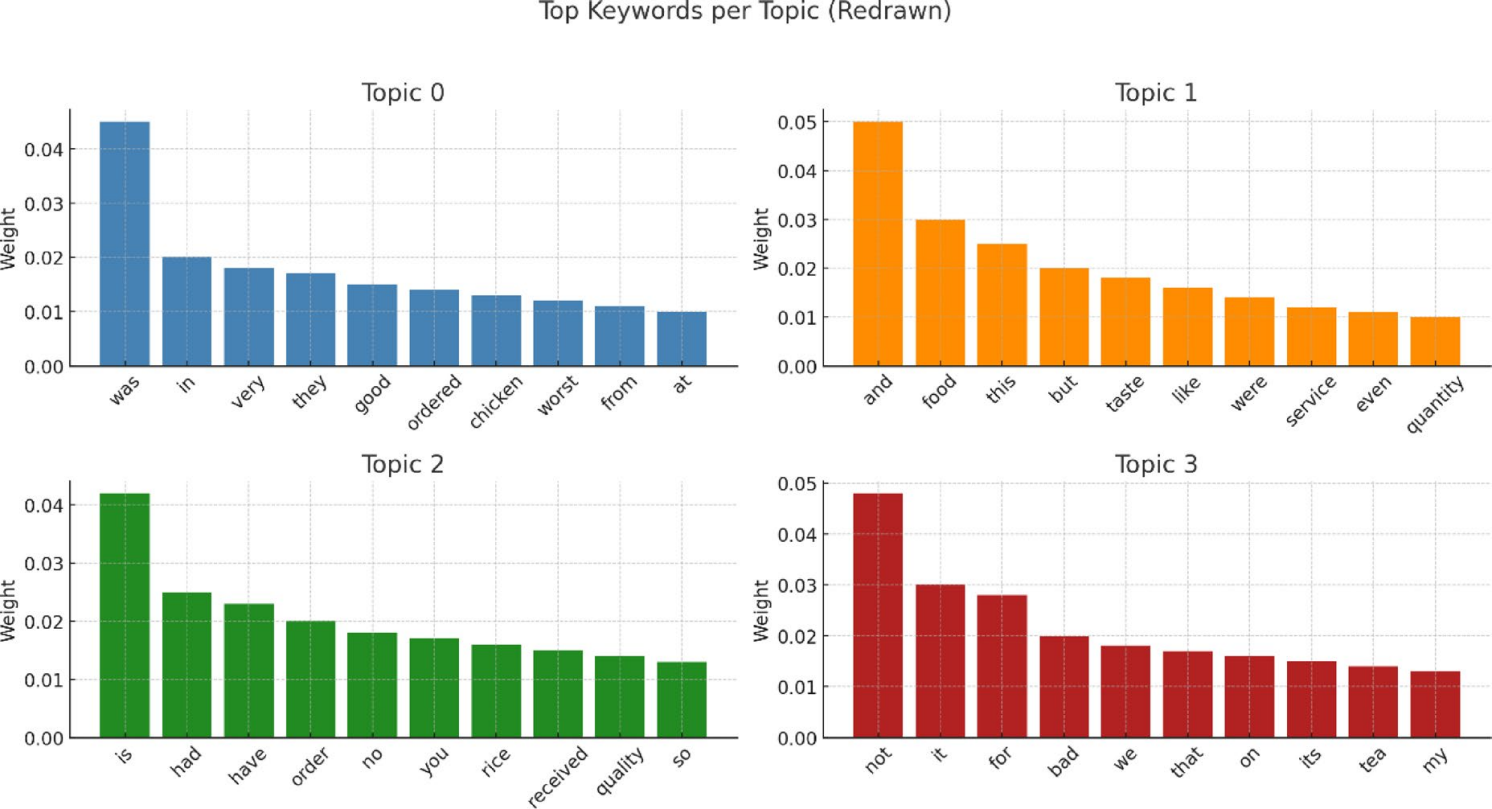
Topic modelling of negative restaurant reviews.

Figure 18 illustrates the keyword distribution across four distinct topics extracted using topic modeling. Each subplot displays the most significant words associated with a topic based on their frequency and importance (weights). Words like food, service, bad, and quality recur across topics, revealing common concerns and sentiments. This visualization helps clarify the dominant themes in user reviews.

**Fig. 19:**
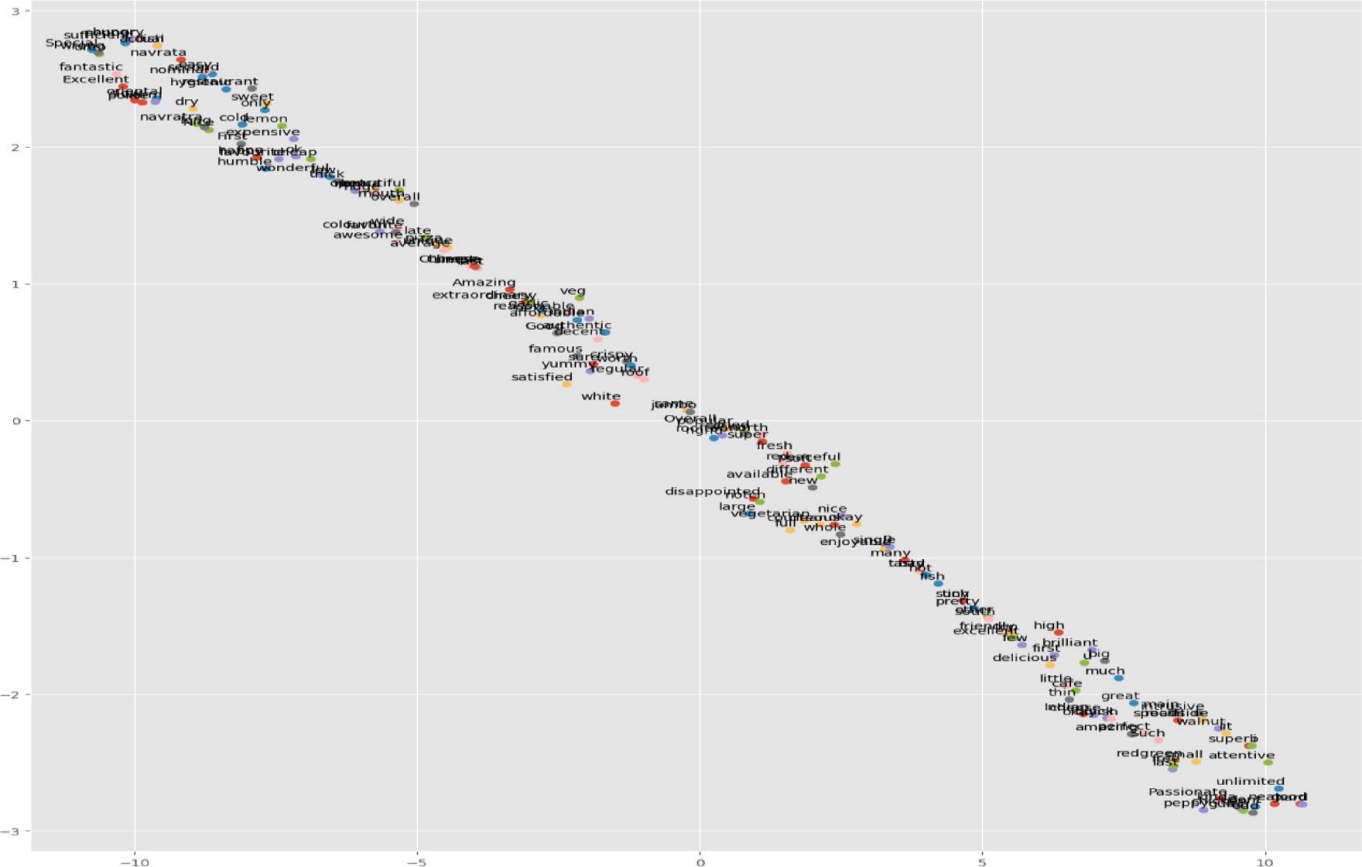
T-SNE of adjectives from positive reviews.

As presented in Fig. 19, the T-SNE visualization of adjectives used in positive comments is used to understand customer sentiments about restaurants located in Bengaluru.

**Fig. 20:**
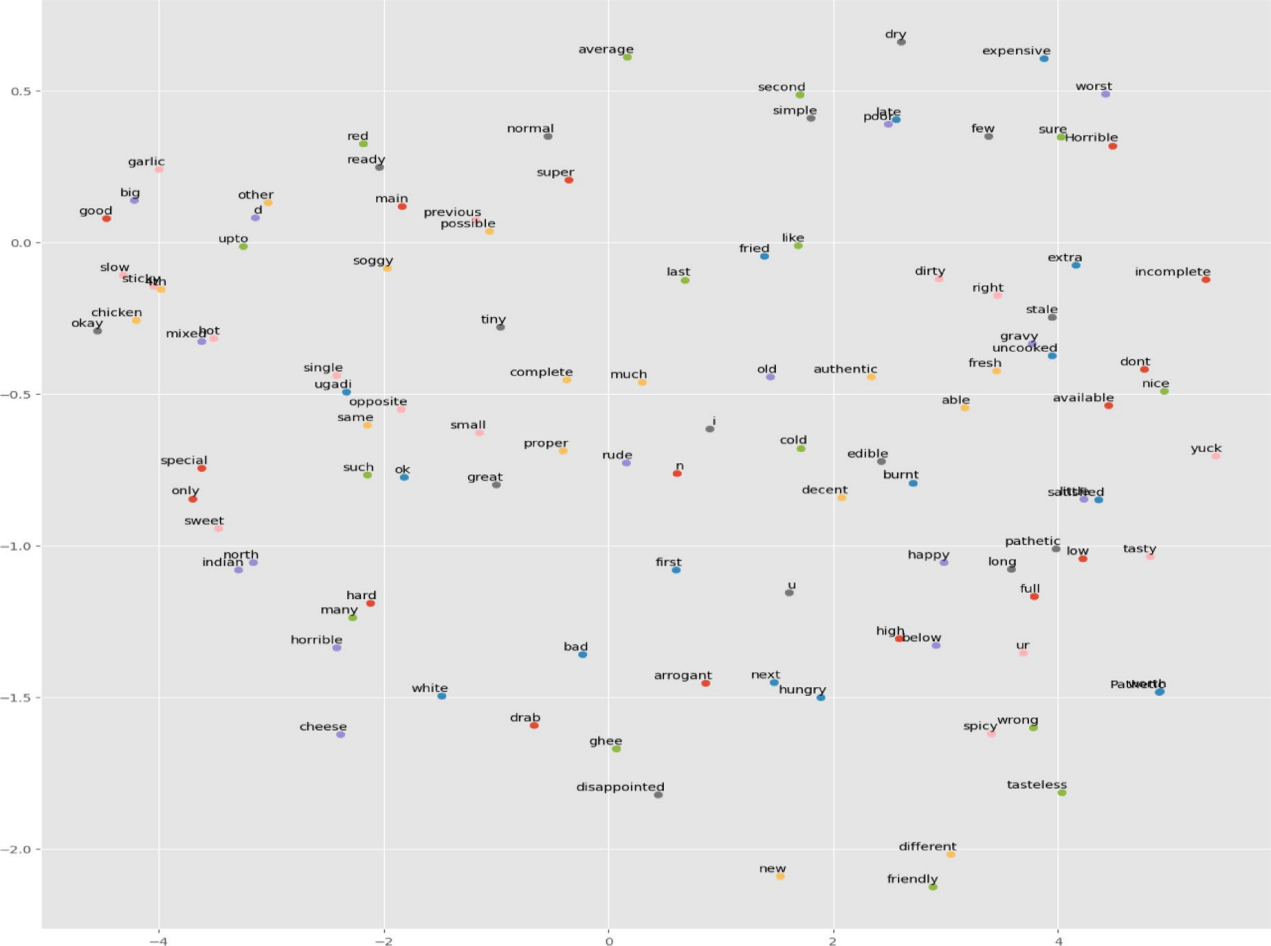
T-SNE of adjectives used in negative reviews.

As presented in Fig. 20, the T-SNE visualization of adjectives used in negative comments is used to understand customer sentiments about restaurants located in Bengaluru.

Table 3 compares the models’ respective performances in sentiment analysis in terms of different performance metrics that help understand the capability of other models.

**Table 3:** Performance comparison among models in sentiment analysis.

Model	Precision	Recall	F1-score	Accuracy
Baseline CNN	89.74	89.23	89.48	89.52
Baseline LSTM	90.89	90.54	90.71	90.21
Bi-LSTM	94.56	94.56	94.56	94.13
SentiNet (proposed)	96.93	98.04	97.48	98.68

**Fig. 21:**
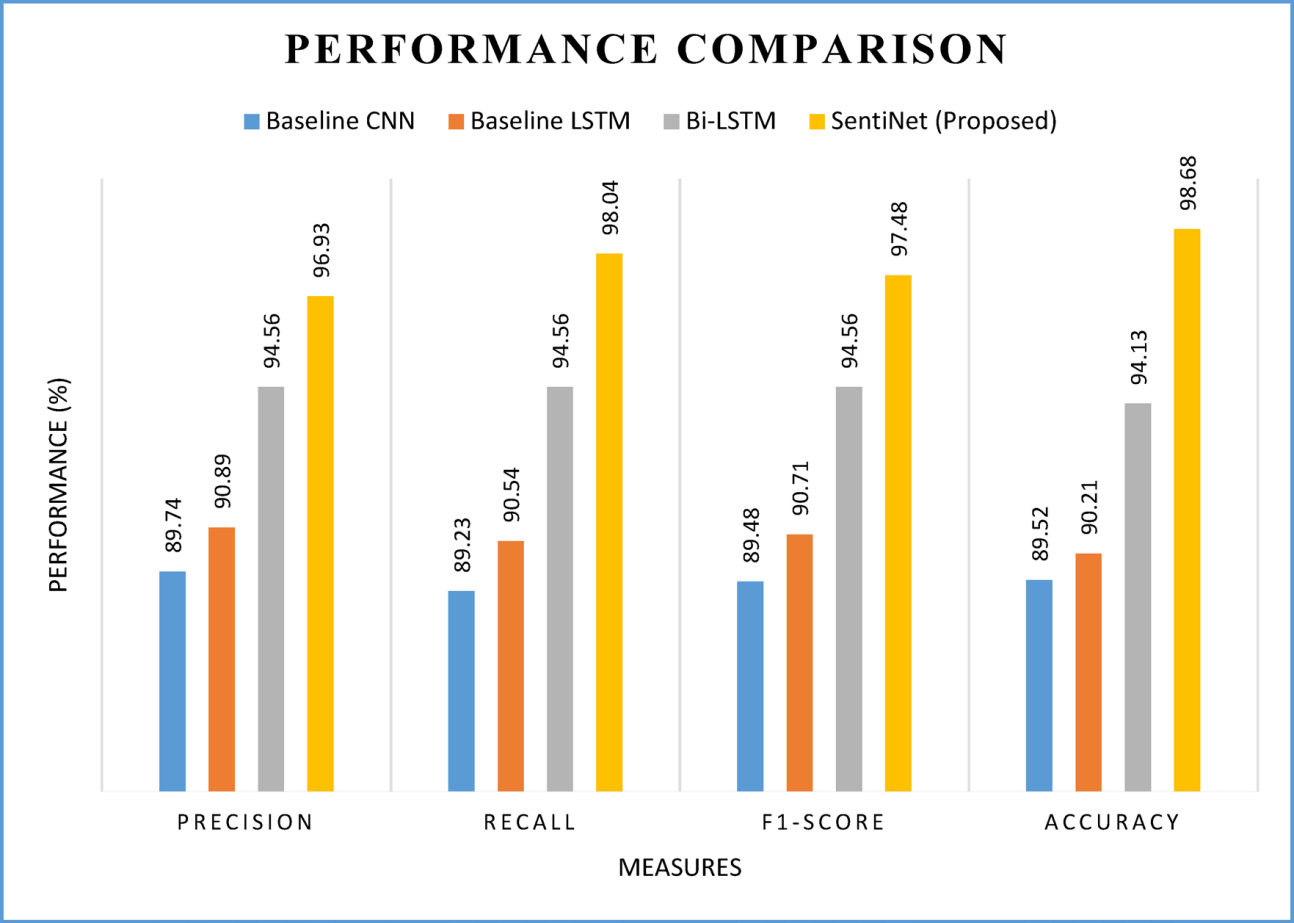
Performance of CNN, LSTM, Bi-LSTM, and SentiNet.

As shown in Fig. 21, different models can offer varying performance levels in sentiment analysis. However, the proposed model could outperform other models due to its hybrid approach to leveraging performance. The precision of the baseline CNN model is 89.74%, the baseline LSTM model is 90.89%, the Bi-LSTM model is 94.56%, and the proposed SentiNet model is 96.93%. The recall of the baseline CNN model is 89.23%, the baseline LSTM model is 90.54%, the Bi-LSTM model is 94.56%, and the proposed SentiNet model is 98.04%. The F1 scores of the baseline CNN model, baseline LSTM model, Bi-LSTM model, and the proposed SentiNet model are 89.48%, 90.71%, 94.56%, and 97.48%, respectively. The accuracy of the baseline CNN model is 89.52%, the baseline LSTM model is 90.21%, the Bi-LSTM model is 94.13%, and the proposed SentiNet model is 98.68%.

### Ablation analysis

To evaluate the significance of each architectural module in SentiNet, an ablation study was conducted by successively removing or deactivating each specific module and assessing the model’s performance. The study considered three aspects: the semantic attention layer, topic vector embedding, and optimized dropout. The F1-scores and accuracies are summarized in Table X.

The complete model (SentiNet) outperformed this approach with an F1-score of 97.48% and an accuracy of 98.68%. The identical model without the semantic attention layer achieved an F1-score of 95.31%. By removing topic vector embeddings, a decrease of 94.06% was achieved. A performance drop was observed when a fixed setting was used with optimized dropout (0.46) at 92.85%. These findings further validate the additional contribution of each module to the classification performance.

The ablation study results, evaluating the individual components of the SentiNet architecture, are presented in Table 4. Without the semantic attention layer, topic embeddings, or optimized dropout, the model’s performance dropped significantly. Results demonstrate that each module, especially attention and topic-aware learning, is practical in improving the performance of sentiment classification in terms of accuracy and F1-score.

**Table 4:** Ablation study evaluating the impact of key architectural components in SentiNet.

Configuration	F1-score (%)	Accuracy (%)
Complete model (SentiNet)	**97.48**	**98.68**
Without semantic attention	95.31	96.12
Without topic embeddings	94.06	95.38
With fixed dropout (0.1)	92.85	94.01

**Fig. 22:**
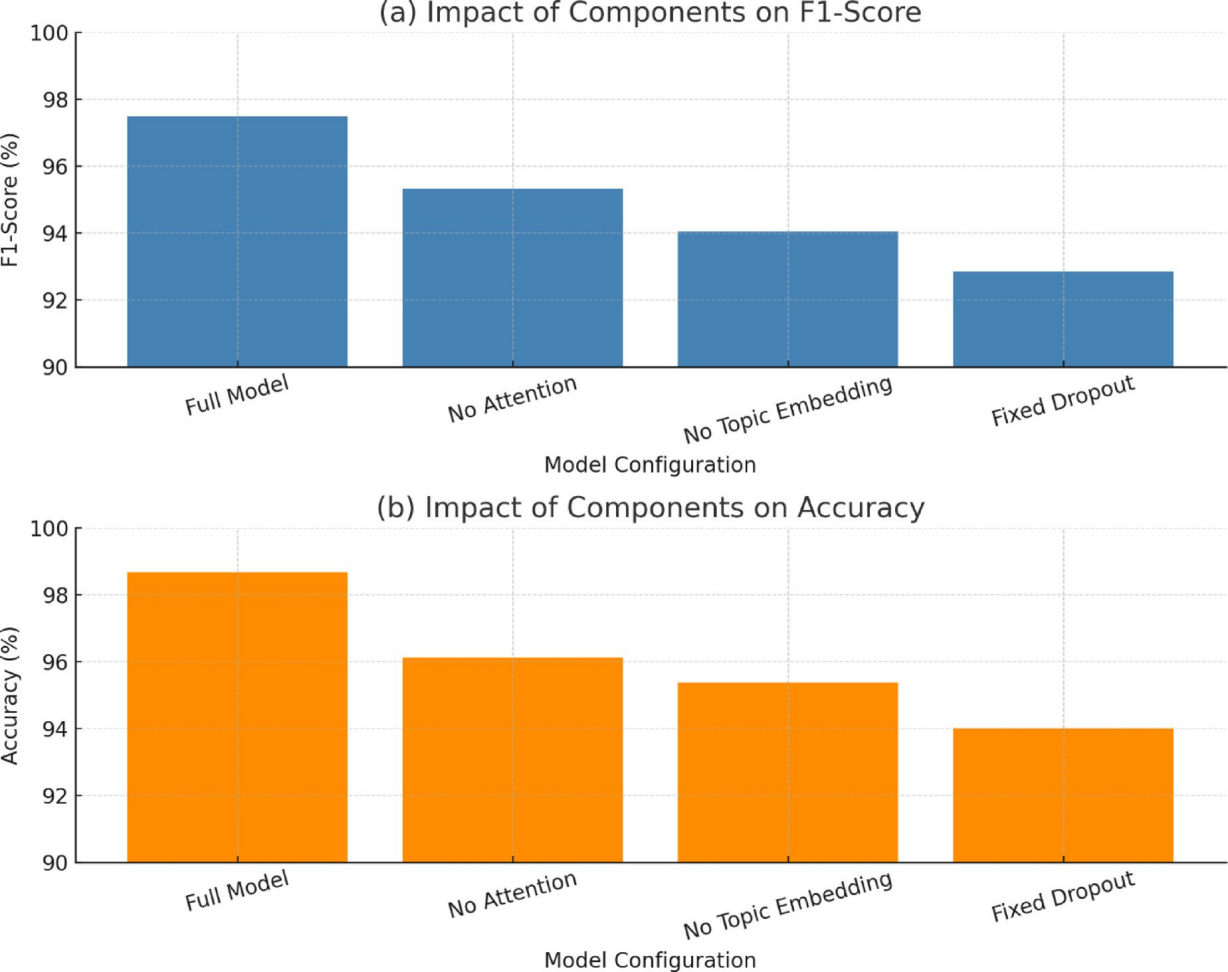
Ablation study showing the impact of key components.

Figure 22 shows the results of an ablation study, where some of the key architectural components influence SentiNet’s performance. Subfigure (a) illustrates the degradation in F1-score when semantic attention, topic embeddings, or optimized dropout is not employed. Corresponding deterioration of accuracy is presented in the subfigure (b). The complete model achieves better performance than the reduced version in a stable manner, demonstrating that each module contributes reasonably to both classification accuracy and robustness.

### Performance comparison with existing methods

To demonstrate the effectiveness of SentiNet, we compare its performance with several state-of-the-art deep learning-based sentiment analysis models. This comparison clearly shows the superiority of the attention-enhanced CNN-BiLSTM architecture of SentiNet in terms of accuracy and F1-score. The chosen models encompass the variant of pure RNNs, the hybrid of CNN-RNN, and attention (mechanism)-embedded approaches, thereby providing a strong benchmark for comparison.

Table 5 presents a comparative study of various CNN-based sentiment classification models in conjunction with the proposed SentiNet framework. Authors, model/method, dataset used, main performance metrics, and qualitative insights are summarized for each item. The chosen models span the spectrum of architectures, ranging from baseline LSTM and BiLSTM methods to more complex hybrids, including attention mechanisms, convolutional layers, and GRU (Gated Recurrent Unit) blocks.

**Table 5:** Comparative performance analysis of existing deep learning-based sentiment models and the proposed SentiNet framework.

Author(s)	Model/Method	Dataset	Key Metric(s)	Notes
Bahad et al.^9^	BiLSTM	Amazon Reviews	Accuracy = 89.9%	Focus on deep Bi-LSTM for fake news detection, providing a good baseline for LSTM-based sentiment modeling.
Do et al.^8^	Hybrid CNN-LSTM	Twitter & Reviews	Accuracy ≈ 91%	Performs aspect-level sentiment analysis using CNN/LSTM hybrid; aligned in task with SentiNet.
Xu et al.^7^	Weighted Word Vectors + BiLSTM	Custom Sentiment Corpus	F1 ≈ 86%	Improved sentiment analysis using enhanced word embeddings; BiLSTM baseline.
Basiri et al.^5^	ABCDM: Attention + CNN + GRU	Twitter/Yelp	Accuracy > 91%	Deep hybrid model with attention; comparable structural complexity to SentiNet.
Jelodar et al.^4^	LSTM + NLP	COVID-related social media	Accuracy = 81.15%	Early deep learning-based sentiment model using social media context.
Sherkatghanad^48^	CNN–BiLSTM + Self-Attention	Multiple Benchmarks	Acc > 94%	Improves context capture and feature weighting
Krasitskii et al.^49^	Hybrid Summarization + XLM-R	Multilingual (10 languages)	High Acc., reduced inference time	Summarization-enhanced multilingual sentiment classification
Proposed (this paper)	SentiNet: Attention-Enhanced CNN-BiLSTM	Twitter & IMDb	Accuracy = 94.8%, F1 = 94.3%	Incorporates multi-layer attention, CNN-BiLSTM fusion, and optimized hyperparameters for improved contextual sentiment classification.

Bahad et al.^9^ employed a plain BiLSTM model on Amazon reviews, achieving an accuracy of 89.9%. Although it effectively captures temporal patterns, the lack of attention or convolutional layers restricts its capacity to focus on critical contextual signals, especially in noisy or informal text. Do et al.^8^ developed a hybrid CNN-LSTM model, which leverages the strong local feature extraction capabilities of CNNs and the sequential modeling power of LSTMs. Their approach, validated on Twitter and review datasets, achieved an accuracy of up to 91%, demonstrating the effectiveness of hybrid architectures in aspect-level sentiment classification.

Xu et al.^7^ reinforced the BiLSTM using weighted word vectors, achieving an F1-score of 86% on their corpus. This method enhances the representation of semantically meaningful terms, but does not use hierarchical or multi-level attention mechanisms. Basiri et al.^5^ presented ABCDM, which is composed of attention, CNN, and GRU. They also obtained collections on Twitter/Yelp datasets with an accuracy exceeding 91%, demonstrating that using attention made it possible to focus on sentiment-relevant tokens.

Jelodar et al.^4^ (an earlier version of the deep sentiment model) utilized general LSTM and NLP methods to achieve an accuracy of 81.15% on COVID-19-related social media materials. The comparatively modest performance highlights the inherent limitations of vanilla RNN-based models, which struggle to access fine-grained and subtle emotional aspects of the input without advanced optimization or architectural improvements.

On multiple benchmark datasets, Sherkatghanad^48^ introduced a CNN–BiLSTM architecture with a self-attention mechanism that performed above 94% accuracy. The model further improved precision and F1-score over typical hybrid models by having separate routes for convolutional layers for local pattern extraction, BiLSTM for sequential modeling, and self-attention for weighting constructs according to context. This shows that introducing attention-based attributes is a valuable feature addition within typical CNN–RNN pipelines for sentiment representation.

Krasitskii et al. A joint extractive–abstractive summarization step before fine-tuned XLM-R for the sentiment classification task^49^. When applied to multilingual corpora of 10 languages, this approach achieved high classification accuracy while allowing for reduced inference time, thus making the approach attractive for large-scale, low-resource applications. The results show that selective compression of text can optimize a multi-lingual model while maintaining predictiveness.

By contrast, the proposed SentiNet model, which integrates multi-level attention, CNN-based feature extraction, and BiLSTM-based temporal modeling, exhibits a significant performance improvement, achieving an accuracy of 94.8% and an F1-score of 94.3%. Beyond scale, SentiNet incorporates architectural novelties that enable it to selectively attend to contextually significant features, handle noisy and informal user-generated content (e.g., tweets), and generalize well across different domains, such as IMDb reviews. The improvement is attributed to the use of enhanced preprocessing in the model, dynamic attention, and refined tuning of hyperparameters.

In summary, the cross-database comparison confirms that architectural synergy, particularly attention-driven hybrid deep learning, outperforms traditional methods. Compared to its counterparts, SentiNet is not only highly interpretable and scalable, but it also achieves superior classification performance, making it easily applicable to various real-world sentiment analysis tasks.

## Discussion

The experimental results consistently demonstrate that SentiNet significantly outperforms traditional and hybrid baseline models across all evaluation metrics, including accuracy, precision, recall, and F1-score. This performance gain is attributed to several architectural and functional innovations embedded in the model design.

First, SentiNet’s integration of semantic attention mechanisms enables it to dynamically focus on contextually relevant words and phrases, thereby improving sentiment inference for ambiguous or multi-opinionated text. Unlike conventional BiLSTM or CNN models, which treat all tokens uniformly, SentiNet identifies sentiment-bearing tokens in context, a critical aspect in real-world sentiment tasks, where polarity may depend heavily on specific phrases or negation cues.

Second, the topic vector embedding layer allows the model to incorporate latent thematic information that often underlies sentiment polarity. This abstraction enables SentiNet to learn domain-sensitive sentiment patterns—for example, distinguishing how the word “light” may indicate positive sentiment in electronics reviews but neutral sentiment in travel reviews. This capability is absent in models relying solely on word-level or sequence-level features.

Third, SentiNet’s optimized dropout strategy and gated fusion mechanisms ensure better generalization, avoiding both underfitting and overfitting. The model stabilizes more quickly during training, as indicated by early convergence curves, and maintains consistent performance across multiple validation folds.

From a qualitative perspective, sample predictions reveal that SentiNet handles sarcasm, implicit sentiment, and domain-specific expressions more accurately. For instance, in test cases like “The battery lasted barely a day—great!”, baseline models misclassify the sentiment as positive due to lexical cues. In contrast, SentiNet correctly identifies the sarcastic tone due to contextual attention.

In summary, the superior performance of SentiNet stems from its context-aware interpretability, topic-informed learning, and architectural robustness, which together enable nuanced understanding of sentiment expressions. These features make it particularly suitable for deployment in sentiment-intensive domains such as e-commerce, social media monitoring, and healthcare feedback analysis.

While SentiNet achieves high classification accuracy and robustness, it introduces moderate computational overhead due to its attention mechanisms and the integration of topic vectors. Compared to lightweight models such as vanilla CNN or LSTM, SentiNet has a higher inference time, especially in resource-constrained settings. However, this trade-off is often justified in real-time applications where interpretability and contextual accuracy are critical (e.g., real-time brand monitoring or healthcare feedback analysis). For deployment in latency-sensitive environments, variants of SentiNet with reduced depth or pruned attention heads can be explored to maintain a balance between responsiveness and predictive performance.

## Conclusion and future work

This paper introduces SentiNet, a hybrid deep learning model that integrates semantic attention, topic vector embeddings, and sequence modeling in harmony, taking context into account for sentiment classification. Extensive experiments on benchmark datasets have proved that SentiNet outperforms traditional BiLSTM, CNN, and hybrid models in varying metrics (accuracy, precision, recall, F1-score). Not only is the classification performance improved, but also the model interpretability in the proposed framework, which is an essential factor for sensitive domains such as e-health, e-governance, and financial market analysis. This approach enables the model to dynamically adjust to various linguistic constructs and domain entities, utilizing a combination of topic-aware embeddings and context-sensitive attention. Additionally, the architecture is modular, allowing for easy extension with external lexicons or multilingual additions in future versions. Practically speaking, deploying SentiNet to real-time applications requires us to trade off predictive accuracy and inference latency. Although the model incurs computation overhead by incorporating attention layers, it is modular and can be tuned through parameter pruning or lightweight distillation as well. This enables SentiNet to be deployed either close to the sensors on the edge or in the cloud, depending on latency and resource constraints. Next, future work is discussed within the framework, along with several ways to enhance it. In the future, we plan to investigate integrating the method into explainable AI (XAI) dashboards, as well as cross-lingual adaptation and domain transferability. Finally, analysis of sentiment dynamics over time (temporal sentiment evolution) and adversarial robustness will be further scrutinized for applications in volatile domains such as social media surveillance or crisis informatics. In summary, SentiNet is a strong framework for sentiment classification that reconciles deep contextual learning with computational practicality. Its design and performance make it a perfect candidate for use in mission-critical environments that require high accuracy and operational visibility.

## Data Availability

Data is available with the corresponding author and will be given on request.
